# Optimizing Peer Distribution of Syphilis Self-Testing Among Men Who Have Sex with Men in China: A Multi-City Pragmatic Randomized Controlled Trial

**DOI:** 10.1007/s10508-022-02507-0

**Published:** 2023-01-10

**Authors:** Yajie Wang, Wei Zhang, Xiao Gong, Jason J. Ong, Michael Marks, Peizhen Zhao, Joseph D. Tucker, Weiming Tang, Dan Wu, Cheng Wang

**Affiliations:** 1 Dermatology Hospital of Southern Medical University, Guangzhou, Guangdong 510091 China; 2 Southern Medical University Institute for Global Health, Guangzhou, Guangdong China; 3 Guangdong Provincial Center for Skin Disease and STI Control, Guangzhou, Guangdong China; 4 University of North Carolina at Chapel Hill, Project-China, Guangzhou, Guangdong China; 5 Department of Biostatistics, Guangzhou Jeeyor Medical Research Co., Ltd, Guangzhou, Guangdong China; 6 Clinical Research Department, Faculty of Infectious and Tropical Diseases, London School of Hygiene and Tropical Medicine, London, UK; 7 Central Clinical School, Monash University, Melbourne, VIC Australia; 8 Hospital for Tropical Diseases, London, UK; 9 Institute for Global Health and Infectious Diseases, School of Medicine, University of North Carolina at Chapel Hill, Chapel Hill, NC USA

**Keywords:** Syphilis, Self-test, Men who have sex with men (MSM), Peer distribution, cRCT, Sexual orientation

## Abstract

Syphilis testing uptake is low among men who have sex with men (MSM) around the world. Syphilis self-testing (SST) may complement facility-based testing; the distribution model is yet to be explored. This study aimed to investigate the effectiveness of peer distribution of syphilis self-testing on promoting syphilis testing. We conducted a three-arm, unblinded, parallel individually randomized controlled trial among MSM in three cities in Guangdong, China. Inclusion criteria were: men who were born biologically male, aged 18 or above, have ever had sex with a man, will refer the interventions to peers, and will take the three-month follow-up survey. Enrolled indexes were randomly assigned in a 1:1:1 ratio into standard-of-care arm (SOC arm), standard SST delivery arm (S-SST arm), and a web-based referral link SST delivery arm (RL-SST arm). The primary outcome was the number of returned photograph-verified syphilis testing results per index. A total number of 300 indexes were enrolled, with 100 indexes in each arm. The number of verified syphilis tests per index conducted by alters was 0.05 in the control arm, 0.51 in the S-SST arm, and 0.31 in the RL-SST arm. The cost per alter tested was $760.60 for SOC, $83.78 for S-SST, and $93.10 for RL-SST. Minimal adverse event was reported among both indexes and alters during the study. This study showed that peer distribution of SST could improve syphilis testing uptake among MSM in China compared to facility-based testing. This approach warrants further consideration as part of expanding syphilis self-testing.

**Supplementary Information:**

The online version contains supplementary material available at 10.1007/s10508-022-02507-0.

## Introduction

Syphilis remains an urgent public health concern globally (Poon et al., 2011). There are approximately 6 million new cases in person aged 15–49 every year around the world (Tsuboi et al., 2021). Men who have sex with men (MSM) are disproportionately affected by syphilis (Chen et al., 2017; Jasek et al., 2017); however, the syphilis testing rate within this population remains low (Wang et al., 2020a). Peer distribution of testing, part of the family of network-based interventions, is one strategy commonly used to expand syphilis testing uptake. This approach allows people to distribute testing referrals to their sexual and/or non-sexual partners (Masters et al., 2016). Peer notification cards (PN) are made available to people at facility-based delivery to promote peer testing (Ong’wen et al., 2020; Wang et al., 2012). However, PN has a low acceptance rate due to fear of social discrimination (Wang et al., 2012), embarrassment (Wang et al., 2020a), and lack of partner contact (Wang et al., 2012; Wang et al., 2020b). In addition, facility-testing services for syphilis have been further restrained during the COVID-19 pandemic (Sentís et al., 2021).

Self-testing can complement facility-based peer testing. In self-testing, an individual collects their own specimen, performs the tests, and interprets the results by themselves (Wang et al., 2021; Wu et al., 2021). Globally, promoting self-testing via peer distribution to expand testing uptake has been increasingly used for human immunodeficiency virus (HIV) (Lu et al., 2020). Past studies have shown that HIV self-testing (HIVST) could increase peer testing (Lightfoot et al., 2018), especially during COVID-19 lockdown (Choko et al., 2021), and helped encourage people in their social network to test for HIV (Masters et al., 2016). No equivalent evaluations have been conducted for syphilis self-testing (SST). A cross-sectional study found that syphilis self-testing is acceptable among MSM in China with minimal harm (Cheng et al., 2020; Wang et al., 2020a, 2020b). Two studies showed that syphilis self-testing could expand syphilis testing uptake among MSM in China (Wang et al., 2022) and Zimbabwe (Sri-Pathmanathan et al., 2022). Syphilis self-testing could decentralize testing and facilitate new service delivery models (Lu et al., 2020). Hence, using peer-based delivery of syphilis self-testing kits to promote testing requires further study.

Current peer distribution models take place in person and may result in a lack of privacy (Zhou et al., 2022), potential for coerced testing (Dovel et al., 2020), and inconvenience (Choko et al., 2019). Some of these problems might be alleviated by online network distribution. This approach allows indexes to send web-based links to their peers and to facilitate more applications for self-testing kits from their peers. It is common for MSM to go on dates and meet people (Wang, 2020) through online platforms because of its anonymity (Li et al., 2012), convenience (Tang et al., 2016) and avoidance of societal stigma (Bien et al., 2015). Distributing kits through online networks could avoid in-person interaction and allow programs to reach a wider population (Lu et al., 2020). In this study, we integrated referral links into peer distribution of syphilis self-testing.

This study aimed to evaluate the effectiveness and cost of peer distribution of syphilis self-testing on increasing syphilis peer testing among MSM compared with standard of care.

## Method

### Participants

The full study protocol has been published previously (Wang et al., 2021). We conducted the study in three cities in Guangdong, China: Dongguan (in a community-based organization), Shenzhen (in a community-based organization), and Foshan (a site in hospital-based STI clinic). All sites were run by MSM community-based organizations (Xinghuo LGBT Center, Shenzhen; Friends Care Center, Foshan; and Rainbow Center, Dongguan) that provide MSM free HIV testing and consultations services. We chose these sites because they had a strong track record of engaging the local MSM community and already provide HIV testing services.

**Fig. 1:**
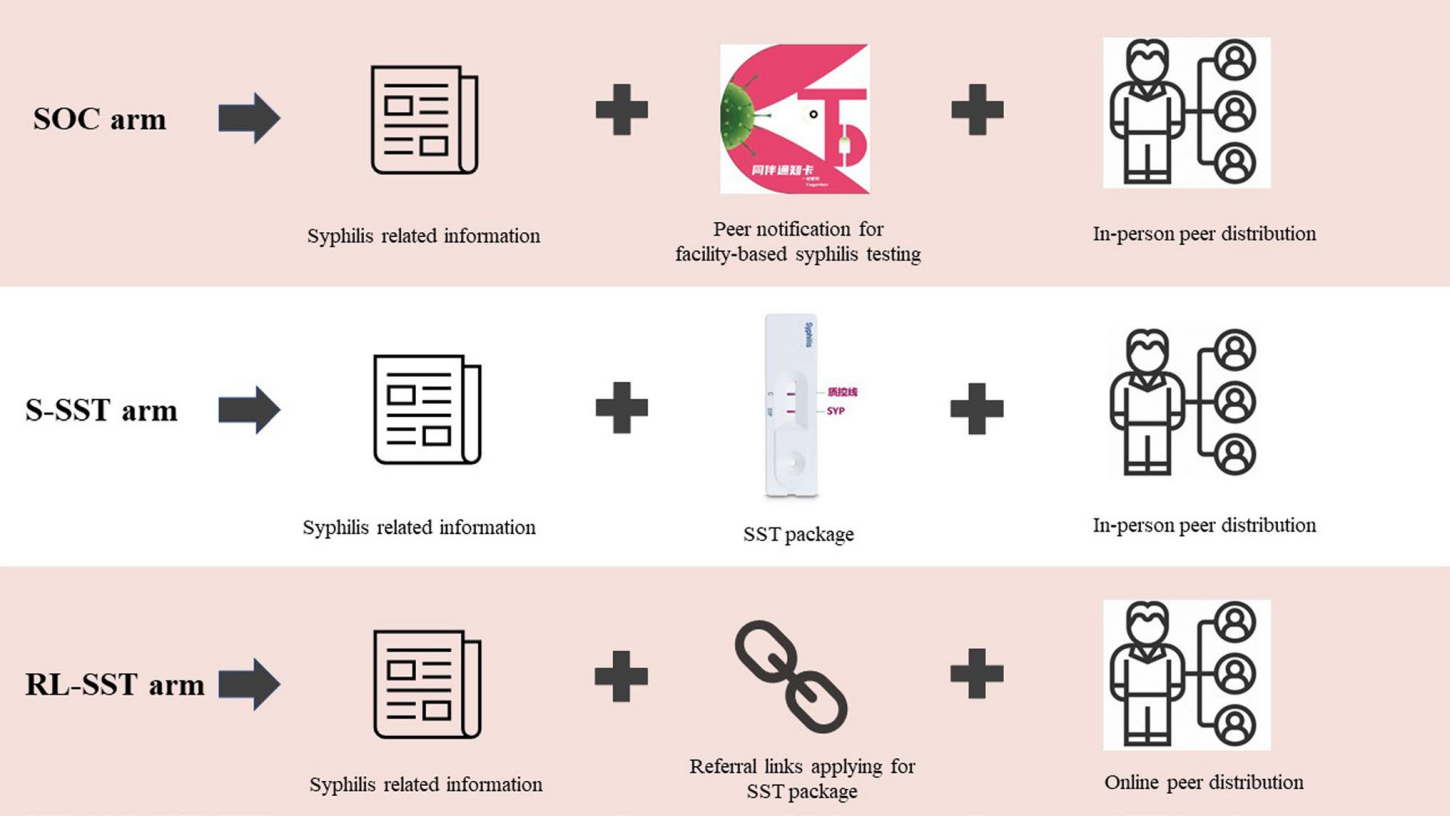
Key concepts of the three study arms. *SOC arm: standard-of-care arm; S-SST arm: standard syphilis self-testing delivery arm; RL-SST arm: referral link syphilis self-testing delivery arm

This was a three-arm, non-blinded, parallel individually randomized controlled trial among MSM. Enrolled indexes were randomly assigned in a 1:1:1 ratio into three arms: standard-of-care arm (SOC arm); standard SST delivery arm (S-SST arm); and a web-based referral link SST delivery arm (RL-SST arm where a referral link is used to apply online for free SST packages) (Fig. 1). The trial procedures were similar for the three arms, all of which included a peer distribution process.

We enrolled index cases who were seeking HIV testing at the study sites by advertising the recruitment in the CBOs’ social media platform, including WeChat (Tencent Inc., Shenzhen, China) group chats and public account, as well as BlueD (Beijing Bluecity Culture and Media Co., Ltd., China). WeChat is the most popular instant messaging application in China, while BlueD is the most popular social networking application among MSM in China. Research staff conducted the recruitment at the partnered organization sites. All participants were asked for their willingness to pass along the information packages or SST packages to their peers and screened for eligibility when they took the survey. Inclusion criteria were: born biologically male, aged 18 or above, have ever had sex with a man, willing to refer the intervention tools to peers, and willing to take the follow-up survey in three months. Eligible participants then entered the survey through a QR code. Digital informed consent was required for every participant at the baseline survey. All participants administered the survey by themselves with assistance from the research staff if needed. We reported our findings according to the Consolidated Standards of Reporting Trials (CONSORT) guidelines (Appendices pp. 18–20).

### Procedure

At each site, eligible indexes were assigned to one of the three arms (1:1:1) using stratified block randomization with a block size of three. It was not possible to mask researchers or participants to group allocation, but the statistician performing the analysis was masked to study arms.

### Interventions

#### Standard-of-Care Arm (Control Arm)

The control arm consisted of a standard peer syphilis testing service delivered through facilities. In this arm, each eligible index MSM initially received three information packages to distribute to social contacts within his network (defined as alters) after completing their baseline survey. Each information package contained: (1) a crowdsourced peer notification card; (2) a health promotion and linkage to care information card; and (3) a syphilis testing result report card. The peer notification card contained healthcare provider location and information, which each alter could use to receive free syphilis screening in a list of nearby facilities in each study site. The health promotion card contained information on the risk of acquiring syphilis, the importance of screening for syphilis, and a link to resources of national syphilis Voluntary Counseling & Testing sites. A result report card contained a QR code that allowed alters to be added to our public WeChat account (as a contact), with which they could scan and upload the photographs of syphilis testing results anonymously and privately and receive online counseling services. Each alter could use a maximum of one package and take one free facility-based syphilis testing service through peer notification card from each index.

#### Standard SST Delivery Arm (S-SST Arm)

Index MSM in this arm initially received three SST packages for distribution after completing the baseline survey. These were accompanied by equivalent health promotion and linkage to care information card and syphilis testing result report cards as in the control arm. Each kit contained equipment for blood sample collection, the syphilis test platform, and a step-by-step pictorial instruction for using the self-test kit. Each index could distribute one SST package to each alter. In this study, we used the syphilis SD Bioline Syphilis 3.0 rapid test kit, whose sensitivity and specificity for syphilis are 85.7–100% and 95.5–99.4%, respectively (Mabey et al., 2006; Unemo et al., 2017).

#### Referral Link SST Delivery Arm (RL-SST Arm)

Index MSM in this arm initially received a web-based identifiable SST referral link instead of physical SST packages, which could be shared with up to three alters after baseline survey. The referral links facilitated a free express delivery of SST packages and collection of the following information from alters: a preferred name (or an alias), a phone number, and post address. Each link would expire after three uses. Each alter could only apply for one SST package with the link, which could be accessed by only one device (both a WeChat account and a phone number).

#### Interventions in All Three Arms

In all three study arms, each package or link was assigned a unique number to allow results tracking and matching to index cases. After testing, all alters were asked to scan the QR code in the result report card to submit photographs of their facility-testing or self-testing results and reported matched number. Then alters were asked to complete an anonymous survey. Regardless of study arm, alters received 3 USD when they returned the syphilis testing result photographs (self-testing or facility-testing results) and completed the study survey. Alters with a reactive result who returned photograph of confirmatory testing results or of a treatment report received another 3 USD. Their matching index also received an extra 3 USD as an incentive for successful distribution. In addition, each index could apply for an additional three packages (refundable 3 USD deposit for each SST package; free of charge for notification card package) or for an additional free referral links when their initial link had been used by three alters returning their test results. In all three arms, each alter who returned a test result could become a “secondary index” if he was willing to and met the following criteria: born biologically male, aged 18 years or above, and had ever had sex with a man. Each secondary index could also apply for three SST packages or information packages with free express delivery in S-SST or control arm, while a referral link in RL-SST arm as primary index for distribution to his alters.

### Measures

All eligible indexes completed a baseline questionnaire at enrollment, which included sociodemographic characteristics, sexual orientations disclosure, number of male sex partners, syphilis testing, HIV testing and other STIs testing, and their size of social network, like “How many MSMs have you known in your live?” Indexes were provided with 3 USD for completing the baseline survey. After uploading the syphilis test result, each alter received an online survey which collected questions on the relationship between the index and the alter, sociodemographic information, sexual behavior, the experience of receiving syphilis testing packages or links, testing history of syphilis, HIV and other STIs, and size of social network. At three-month follow-up, we invited all indexes to complete a brief online questionnaire that collected information on their experience of distributing syphilis testing packages or links, relationship between the indexes and the alters, reasons of unwillingness to distribute, history of syphilis, HIV and other STIs testing in the past three months; and sexual behaviors in the past three months. Potential adverse events such as forced testing, physical and/or verbal abuse, or causing mistrust from alters during the delivery procures were both asked from indexes and alters. Indexes were provided with 4 USD for completing the follow-up survey.

#### Follow-Up Support

A syphilis counselor was available to support through WeChat and telephone from 8:00 AM to 5:30 PM, Monday through Friday. Support included giving pretest counseling, instructing how to use the self-test kit, helping to interpret results, and providing advice for reactive test results. Alters with a reactive self-testing results were referred to undergo a free confirmatory laboratory testing and clinical examination at a designated clinics or hospitals. A research assistant undertook further follow-up to obtain confirmatory testing results and treatment information for men diagnosed with syphilis.

#### Outcomes

The primary outcome was the number of alters who returned photograph-verified syphilis testing results per index, including both facility-based tests and self-tests, over a three-month period. The verification process was conducted by a research assistant by checking the uploaded test result against the result report card using a standard verification protocol (shown in Appendices pp. 6–7). Secondary outcomes included the proportion of first-time syphilis testing among participants, proportion of testers with a positive syphilis testing result, and adverse events during the delivery procedures in each arm during the trial. The secondary outcomes were assessed based on returned syphilis testing result photographs or self-reported data from alter survey. We also report the total economic cost, cost per person tested, and cost per person managed for syphilis in each arm. The costs were collected from a healthcare provider perspective and reported in 2021 US dollars.

### Statistical Analysis

A detailed sample size calculation is provided in the Appendix (p. 15). Overall, we calculated 300 indexes required to assess our primary outcome. We report descriptive statistics for sociodemographic and behavioral characteristics in each study arm. All inferential tests were two-sided with a type 1 error level of 0.05. For the primary outcome, we put the number of alters, per index, who provided a verified syphilis test as dependent variable, and intervention arm as independent variable using a negative binomial regression model. Relative risks (RR) and corresponding 95% confidence intervals (CIs) were estimated for the number of alters returning a result per index between each arm. We also conducted a subgroup analysis stratified by age, number of male partners in the past three months, number of MSM that indexes have known in their lives, and self-testing experience of syphilis.

The secondary outcomes compared the differences in the proportion of first-time syphilis tested alters, alters with syphilis-reactive results, and reported adverse events among alters. We applied logistic regression modelling to estimate the odds ratio (OR) and corresponding 95% CIs for proportion differences between arms. We also compared the differences on characteristics of indexes who completed follow-up survey or not. All analyses were conducted using SAS version 9.4 (SAS Institute, Cary, NC).

We estimated the economic cost of syphilis testing in the three study arms. Costs were categorized as fixed or variable costs. Fixed cost referred to items that were independent of the number of tests conducted, including cost of start-up, building rent and office equipment. Variable cost referred to items that were dependent on the number of tests conducted, including SST kits, standard-of-care testing supplies, and personnel time. Costs related to personnel were calculated by multiplying the staff time associated with each program activity by their hourly wage. We also calculated the cost per alter tested and the cost per alter diagnosed with syphilis. We calculated and ranked the incremental cost-effectiveness ratios (ICERs) to identify the most cost-effective intervention.

We followed the intention-to-treat principle and did not have missing data in terms of primary and secondary outcomes. A data monitoring committee was not used as the trial risks were deemed minor. This study was registered in the Chinese Clinical Trial Registry (ChiCTR2000036988). These study findings were reported according to the extension of the CONSORT 2010 statement.

## Results

### Participant Recruitment and Flow

**Fig. 2:**
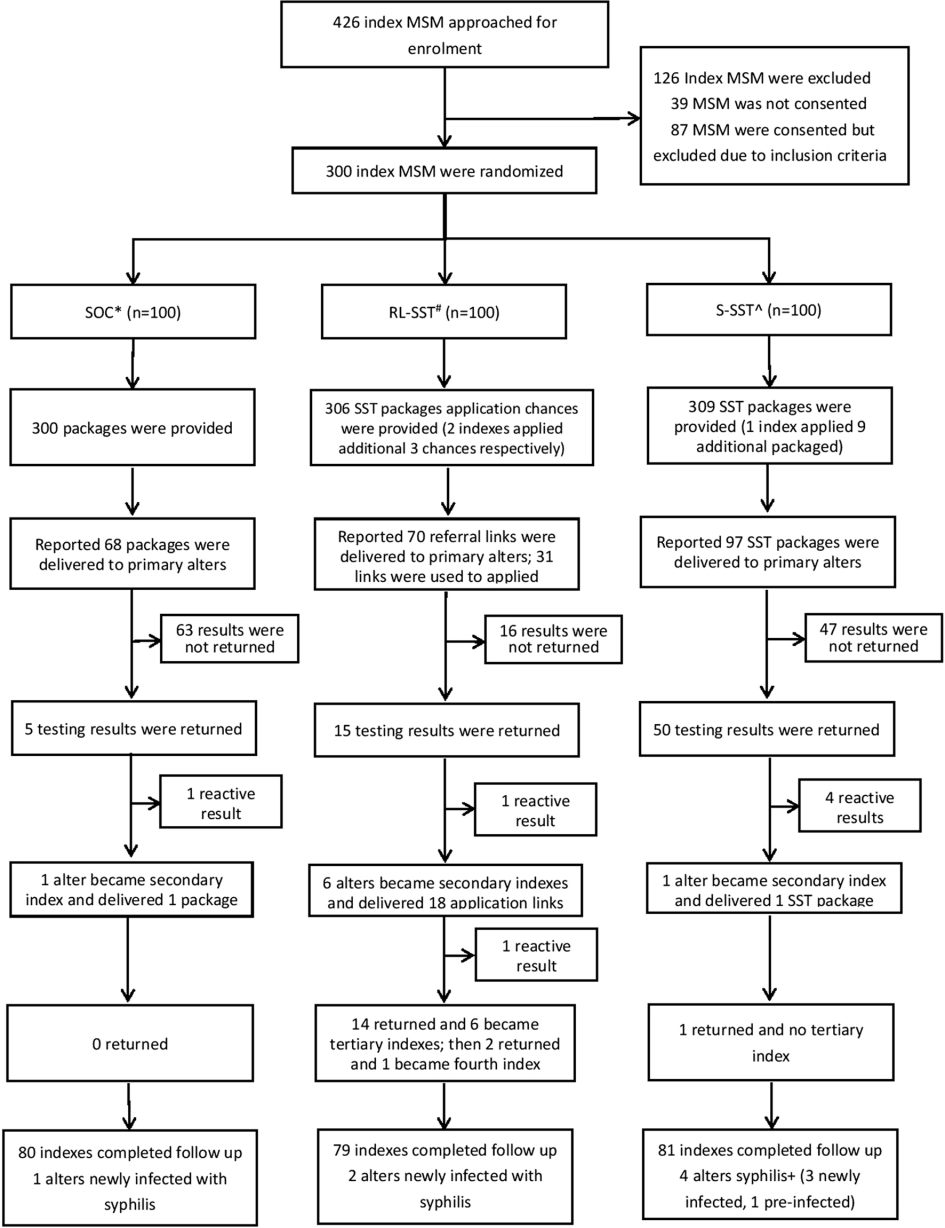
Trial flow chart

Study data were collected from November 19, 2020, to August 12, 2021. Overall, 426 index MSMs were approached for enrollment and clicked the baseline survey link; 39 men declined to provide consent, and 387 were screened for study eligibility (Fig. 2). In total, 87 men were excluded because they were younger than 18 years old (*n* = 35), were not born biologically male (*n* = 1), reported no sex with men (*n* = 22), stated they would not pass along the information packages or SST packages to alters (*n* = 11), or would not complete follow-up survey in three months (*n* = 18).

A total of 300 men were enrolled and randomly assigned to one of the three arms: 100 men in the standard-of-care arm, 100 men in the S-SST arm, and 100 men in the RL-SST arm (Fig. 2). After three months, 80 indexes in the SOC arm, 81 indexes in the S-SST arm, and 79 indexes in the RL-SST arm completed the follow-up survey, respectively. Baseline characteristics of study participants stratified by loss-to-follow-up is shown in Appendix (p. 11).

### Participant Characteristics

Baseline characteristics were similar across the three arms (Table 1). Around half the participants were 30 years of age or younger (52.0%). About half of indexes reported that they had multiple male sexual partners in the past three months (55.3%), and known more than two MSMs in their social network (48.0%). In total, 211 (70.3%) had ever previously tested for syphilis, but only 37.4% (79/211) had previously used a syphilis self-test. Overall, 274 (91.3%) indexes had ever tested for HIV and 57.3% (157/274) had used HIV self-testing. There were no significant differences in demographic characteristics except in number of male sex partners in the past 3 months (*p* = 0.020) and in ever infected with HIV or not (*p* < 0.001) (Table 1).

**Table 1:** Baseline characteristics of indexes in three arms

Characteristics	Total (*N* = 300)	SOC arm (*N* = 100)	S-SST arm (*N* = 100)	RL-SST arm (*N* = 100)
*Age* (in years)				
≤ 30	144 (48.0)	56 (56.0)	40 (40.0)	48 (48.0)
> 30	156 (52.0)	44 (44.0)	60 (60.0)	52 (52.0)
*Marital status*				
Ever married	96 (32.0)	26 (26.0)	36 (36.0)	34 (34.0)
Never married	204 (68.0)	74 (74.0)	64 (64.0)	66 (66.0)
*Highest education*				
High school or below	158 (52.7)	51 (51.0)	51 (51.0)	56 (56.0)
College or above	142 (47.3)	49 (49.0)	49 (49.0)	44 (44.0)
*Annual income (USD, $)*				
≤ 5500	47 (15.7)	16 (16.0)	23 (23.0)	8 (8.0)
5501–15,000	199 (66.3)	67 (67.0)	58 (58.0)	74 (74.0)
> 15,000	54 (18.0)	17 (17.0)	19 (19.0)	18 (18.0)
*Sexual Orientations Disclosure*				
Yes	254 (84.7)	82 (82.0)	88 (88.0)	84 (84.0)
No	46 (15.3)	18 (18.0)	12 (12.0)	16 (16.0)
*Number of male sex partners in the past 3 months*				
≤ 1	166 (55.3)	60 (60.0)	44 (44.0)	62 (62.0)
> 1	134 (44.7)	40 (40.0)	56 (56.0)	38 (38.0)
*Number of MSM that indexes have known in their lives*				
≤ 2	156 (52.0)	48 (48.0)	50 (50.0)	58 (58.0)
> 2	144 (48.0)	52 (52.0)	50 (50.0)	42 (42.0)
*Ever tested for HIV*				
Yes	274 (91.3)	90 (90.0)	92 (92.0)	92 (92.0)
No	26 (8.7)	10 (10.0)	8 (8.0)	8 (8.0)
*Ever self-tested for HIV*				
Yes	157 (57.3)	56 (62.2)	54 (58.7)	47 (51.1)
No	117 (42.7)	34 (37.8)	38 (41.3)	45 (48.9)
*Ever infected with HIV*				
Yes	32 (10.7)	6 (6.0)	21 (21.0)	5 (5.0)
No	268 (89.3)	94 (94.0)	79 (79.0)	95 (95.0)
*Ever tested for syphilis*				
Yes	211 (70.3)	74 (74.0)	66 (66.0)	71 (71.0)
No	89 (29.7)	26 (26.0)	34 (34.0)	29 (29.0)
*Ever self-tested for syphilis*				
Yes	79 (37.4)	31 (41.9)	25 (37.9)	23 (32.4)
No	132 (62.6)	43 (58.1)	41 (62.1)	48 (67.6)

SOC arm: standard-of-care arm; S-SST arm: standard syphilis self-testing delivery arm; RL-SST arm: referral link syphilis self-testing delivery arm

### Peer Testing

For the primary outcome, the number of verified syphilis tests per index conducted by alters was 0.05 (5/100) in the control arm, 0.51 (51/100) in S-SST arm, and 0.31 (31/100) in RL-SST arm. Compared to the control arm, both the S-SST (RR = 10.2, 95% CI:2.9–36.3) and the RL-SST arm (RR = 6.2, 95% CI 1.7–22.5) were associated with a significantly higher number of tested alters per index. Although no significant difference was found on the overall number of tested alters per index between S-SST and RL-SST arm, there were more primary alters in S-SST arm than in RL-SST arm (Table 2).

**Table 2:** Primary outcomes analysis at the end of the study

	Number of alters motivated per index (*n*/*N*)			RR (95% CI)	*p* value	RR (95% CI)	*p* value	RR (95% CI)	*p* value
	SOC	S-SST	RL-SST	S-SST versus SOC		RL-SST versus SOC		S-SST versus RL-SST	
Overall	0.05 (5/100)	0.51 (51/100)	0.31 (31/100)	10.2 (2.9, 36.3)	< 0.001***	6.2 (1.7, 22.5)	0.006**	1.7 (0.6–4.4)	0.321
Primary level	0.05 (5/100)	0.5 (50/100)	0.15 (15/100)	10.0 (3.1, 32.0)	< 0.001***	3.0 (0.9, 10.3)	0.082	3.3 (1.3–8.3)	0.010**
Subgroup analysis									
*Age*									
≤ 30	0.0 (2/56)	0.8 (31/40)	0.2 (9/48)	21.7 (3.4, 139.9)	0.001**	5.3 (0.8, 35.4)	0.088	4.1 (1.0–17.5)	0.054
> 30	0.1 (3/44)	0.3 (20/60)	0.4 (22/52)	4.9 (0.9, 27.1)	0.069	6.2 (1.1, 35.2)	0.039*	0.8 (0.2–2.9)	0.720
*Number of male partners in the past 3 months*									
≤ 1	0.1 (4/60)	0.7 (30/44)	0.5 (29/62)	10.2 (2.1, 50.0)	0.004**	7.0 (1.6, 31.8)	0.012*	1.5 (0.4–5.3)	0.567
> 1	0.0 (1/40)	0.4 (21/56)	0.1 (2/38)	15.0 (1.6, 144.1)	0.019*	2.1 (0.2, 30.1)	0.583	7.1 (1.2–43.0)	0.032
*Number of MSM that indexes have known in their lives*									
≤ 2	0.0 (2/48)	0.6 (31/50)	0.4 (26/58)	14.9 (2.2, 100.1)	0.006**	10.8 (1.6, 70.8)	0.014*	1.4 (0.4–5.1)	0.628
> 2	0.1 (3/52)	0.4 (20/50)	0.1 (5/42)	6.9 (1.4, 34.8)	0.019*	2.1 (0.3, 12.7)	0.434	3.4 (0.8–14.9)	0.111
*Ever self-tested for syphilis*									
Yes	0.1 (3/31)	0.7 (17/25)	1.0 (24/23)	7.0 (0.6, 82.8)	0.121	10.8 (0.9,131.1)	0.062	0.7 (0.1–7.1)	0.725
No	0.1 (2/43)	0.5 (21/41)	0.1 (4/48)	11.0 (2.1, 58.5)	0.005**	1.8 (0.3, 11.7)	0.542	6.2 (1.6–23.4)	0.008**

RR: relative ratio; CI: confidence interval; SOC: standard-of-care; S-SST: standard syphilis self-testing delivery; RL-SST: referral link syphilis self-testing delivery

Figure 2 summarizes the distribution of testing kits and referrals across the three arms. In the SOC arm, 80 indexes completed a follow-up survey and nearly half (38/80) reported they had delivered packages to primary alters. Five primary alters returned testing results and one (20%) became secondary index. In the S-SST arm, 81 indexes completed follow-up and more than 60% (50/81) reported they had delivered packages to primary alters. Fifty primary alters returned results and 2% became secondary index. In the RL-SST arm, 79 indexes completed follow-up and more than half (42/79) reported delivering packages to primary alters. Fifteen primary alters returned results, and 40% became secondary indexes. A total of six tertiary indexes and one fourth-level indexes were observed. There was significant difference on number of alters who became secondary indexes among three arms (*p* < 0.001). More information regarding the peer distribution of syphilis testing is listed in Appendix Figure S5 (p. 10).

The impact of the interventions varied by indexes’ age, number of male partners in the past three months, social network size, and syphilis self-testing history. Compared with the SOC arm, the S-SST arm increased the effect of peer testing in MSMs aged 30 years or less (RR = 21.7, 95% CI 3.4–139.9), amongst indexes with both one or less male partners (RR = 10.2, 95% CI 2.1–50.0) and indexes with multiple male partners (RR = 15.0, 95% CI 1.6–144.1) in the past three months, amongst indexes with smaller (RR = 14.9, 95% CI 2.2–100.1) and larger (RR = 6.9, 95% CI 1.4–34.8) MSM social circles, and amongst indexes who had never self-tested for syphilis (RR = 11.0, 95% CI 2.1–58.5). By contrast, RL-SST increased the effect of peer testing in MSMs aged more than 30 years (RR = 6.2, 95% CI 1.1–35.2), indexes who had one or less male partners (RR = 7.0, 95% CI 1.6–31.8), or who had a smaller MSM social circle (RR = 10.8, 95% CI 1.6–70.8) (Table 2).

### Secondary Outcomes

Table 3 describes the secondary outcomes in three arms. The proportion of first-time syphilis testers among alters was 20.0% (1/5) in the control arm, 56.5% (26/46) in S-SST arm, 67.7% (21/31) in RL-SST arm. In the SOC arm, one new infected case was detected from alters and reported they had been treated in the study period. In S-SST arm, one past and three new cases were detected, and all new cases reported they had been treated. In RL-SST arm, two new cases were detected, and all reported they had been treated. The proportions of adverse events during distribution procedures reported from both alters (SOC: 20.0% (1/5); RL-SST: 29.0% (9/31); S-SST: 26.1% (12/46)) and indexes (SOC: 51.4% (19/37); RL-SST: 54.8% (23/42); S-SST: 39.6% (19/48)) did not vary significantly between three arms. There were 6.3% (8/127) of indexes and 10% (8/82) alters reporting experiencing verbal abuse, physical abuse or forced testing during the distribution procedures.

**Table 3:** Secondary outcomes analysis at the end of the study

Secondary outcomes	Proportion (%, *n*/*N*)			OR (95% CI)	*p* value	OR (95% CI)	*p* value	OR (95% CI)	*p* value
	SOC	S-SST	RL-SST	S-SST versus SOC		RL-SST versus SOC		S-SST versus RL-SST	
First-time syphilis testers among motivated index	20.0 (1/5)	56.5 (26/46)	67.7 (21/31)	5.2 (0.5–50.2)	0.154	8.4 (0.8–85.2)	0.072	0.6 (0.2–1.6)	0.324
Alters with syphilis-reactive results	20.0 (1/5)	7.8 (4/51)	6.5 (2/31)	0.3 (0.0–3.8)	0.382	0.3 (0.0–3.8)	0.335	1.2 (0.2–7.2)	0.815
Identified adverse events among alters	20.0 (1/5)	26.1 (12/46)	29.0 (9/31)	1.4 (0.1–13.9)	0.768	1.6 (0.2–16.7)	0.678	0.9 (0.3–2.4)	0.776
Identified adverse events among indexes	51.4 (19/37)	39.6 (19/48)	54.8 (23/42)	0.6 (0.3–1.5)	0.281	1.1 (0.5–2.8)	0.762	0.5 (0.2–1.3)	0.152

Five alters in S-SST arm refused to answer the alter questionnaire

SOC: standard-of-care; S-SST: standard syphilis self-testing delivery; RL-SST: referral link syphilis self-testing delivery; OR: odds ratio; CI: confidence interval

### Cost-Effectiveness

Table 4 summarizes the economic evaluation: the cost per alter tested was $760.60 for SOC, $83.78 for S-SST, and $93.10 for RL-SST. The cost per alter newly diagnosed with syphilis was $3803 for SOC, $1424 for S-SST, and $1443 for RL-SST. For ICER, RL-SST was cheaper and more effective than SOC in terms of cost per additional alter tested, and the ICER for S-SST compared to RL-SST was $69.35 per additional alter tested (More details of cost items are included in the model in Table S3, Appendices p. 13–14).

**Table 4:** Incremental cost-effectiveness ratios of cost per alter tested and cost per alter diagnosed

	Cost	Incremental cost	Effectiveness	Incremental effectiveness	ICER
			*Number of alters tested*		
RL-SST	2886		31		
SOC	3803	917	5	− 26	Dominated^a^
S-SST	4273	1387^b^	51	20^#^	69.35
			*Number of alters newly diagnosed*		
RL-SST	2886		2		
SOC	3803	917	1	− 1	Dominated^a^
S-SST	4273	1387^b^	3	1	1387
			*% of alters tested*		
RL-SST	2886		0.094		
SOC	3803	917	0.007	− 0.087	Dominated*
S-SST	4273	1387^b^	0.088	− 0.006^#^	Dominated*
			*% of alters diagnosed*		
RL-SST	2886		0.001		
SOC	3803	917	0.006	0.005	$183,400
S-SST	4273	1387^b^	0.007	0.001	$1,387,000

*ICER* incremental cost-effectiveness ratio, *RL-SST* referral-link syphilis self-testing arm, *SOC* standard-of-care arm, *S-SST* standard syphilis self-testing delivery arm

^a^The comparator was cheaper and more effective

^b^Compared with the next best option, i.e., RL-SST

## Discussion

In this study, we found that peer distribution of syphilis self-testing improved syphilis testing uptake among MSM compared to standard-of-care. We evaluated different models for peer testing and found that person-to-person distribution resulted in a greater uptake of primary alter testing than a peer-led online referral system. To our knowledge, this is the first study to evaluate different models for syphilis peer testing and provides important insights into how to further increase uptake of syphilis testing among MSM in China.

Our study showed that peer distribution of syphilis self-testing kits can improve syphilis testing uptake compared to referral for facility-based testing. We observed that indexes and alters in both intervention arms were more likely to provide test kits to their peers than those in the standard-of-care arm. This is consistent with the results in studies of secondary distribution of HIVST in China (Sha et al., 2022; Zhou et al., 2022) and in Uganda (Okoboi et al., 2020). We found a higher distribution rate for face-to-face distribution of self-testing kits (S-SST) than the other two arms and a higher rate of returned verified test kits amongst alters in this study arm at primary level. This suggest that face-to-face distribution was widely acceptable in our study population. In this study, we directly provided syphilis self-testing kits to indexes of S-SST arm at recruitment, during which we gave them instructions on how to pass along the testing kits to their peers. This might facilitate easier face-to-face distribution in this arm. This finding differs from the findings in studies on peer distribution of HIVST, which required indexes to order self-testing kits themselves (Lu et al., 2020; Wu et al., 2021). We did note that the RL-SST arm resulted in a higher numbers of peers being reached compared to the S-SST arm, which aligns with some literature on online peer referral resulting in wider coverage for HIVST (Zhou et al., 2022). Subgroup analysis results showed that indexes who were 30 years old or below were more likely to pass along the syphilis testing kits to their peers when compared with standard of care. However, men who aged above 30 preferred distribute syphilis self-testing using referral links. Among indexes who never used syphilis self-testing, those in S-SST arms distributed more testing packages than those in RL-SST and SOC arms. Future research on assessing types of indexes, alters and relationship characteristics that predict sharing more kits is needed.

Our study observed a relatively low rate of adverse outcomes among both indexes and alters during the study. The frequency of coerced testing, physical and verbal abuses among alters was low, which is consistent with studies on secondary distribution of HIVST in Malawi (Choko et al., 2021), Kenya (Ong’wen et al., 2020), and Tanzania (Conserve et al., 2018). Other adverse events such as being misunderstood, mistrusted, or alienated were occasionally reported (Appendices p. 16–17). This may reflect the fact that many MSM have limited knowledge about syphilis self-testing and stigma against syphilis testing continues to exist (Turpin et al., 2020). Enhancing education on syphilis as well as syphilis self-testing among MSM community is needed to tackle this problem.

Previous economic evaluation on syphilis self-testing showed that syphilis self-testing is cheaper than facility-based testing (Wang et al., 2022). Our economic evaluation assessed the cost of different distribution approaches. We found that face-to-face distribution of syphilis self-testing kits had a lower cost per alter tested compared to both SOC and RL-SST arms. Unlike the S-SST arm, where we gave out the SST kits at recruitment sites, postal fees were generated when alters applied for syphilis self-testing kits with referral links. Our finding on costing provides an important reference and strategy for healthcare staff and related stakeholders when planning interventions and promoting peer testing of syphilis and/or other STIs among key populations.

This study has implications for policy and implementation. First, given that many facilities providing syphilis testing were completely or partially closed during COVID-19 restrictions, our study offers an innovative approach to distribute syphilis self-testing. Second, this study captures the identifiers of key indexes who could disseminate syphilis self-testing in social network among MSM. With this reference, health workers can tailor syphilis peer testing services among key populations. Furthermore, this study has laid the foundation for exploring novel approaches of peer distribution. Future studies should examine the index-alters social network ties and alters characteristics that predict higher distribution and acceptability of syphilis self-testing. Lastly, peer distribution of standard syphilis self-testing has lower costs for each alter tested. With the cost reduction of self-testing and minimal harm caused, we can extend this approach to other STI peer testing among key population in LMICs.

Our study has several limitations. First, people’s social activities have been largely restricted due to COVID during our study period, which brought challenges to both recruitment and distribution amongst peer networks. Second, recruitment took place in specialist’s clinics, CBOs’ offices, and outreach venues. As such, our study might potentially exclude people who were less likely to connect with the CBOs. Lastly, we used a syphilis rapid test instead of the HIV/Syphilis rapid dual test used in other studies (Cheng et al., 2020; Wang et al., 2020a) to avoid contamination from HIV testing, but this might have discouraged people who also wanted to test for HIV.

In conclusion, this RCT assessed the effectiveness of peer distribution of syphilis self-testing. The findings show that peer distribution of syphilis self-testing could increase syphilis testing uptake among MSM and provide evidence to help optimize syphilis peer testing services among key populations. This approach warrants further consideration as part of expanding syphilis self-testing.

## Supplementary Information

Below is the link to the electronic supplementary material.

Supplementary file1 (DOCX 3000 KB)

## Data Availability

The datasets used and/or analyzed during the current study are available from the corresponding author, Cheng Wang, Email: wangcheng090705@gmail.com, on reasonable request.
