# Supplementary material for: Optimizing Peer Distribution of Syphilis Self-Testing Among Men Who Have Sex with Men in China: A Multi-City Pragmatic Randomized Controlled Trial
Source: Arch Sex Behav. 2023 Jan 10;52(5):2025–36. doi: 10.1007/s10508-022-02507-0 (PMC9831370; doi:10.1007/s10508-022-02507-0)
Supplement: Supplementary file 1 — Supplementary file1 (DOCX 3000 KB) [file 10508_2022_2507_MOESM1_ESM.docx]

# Appendix

[Appendix 1](#_Toc101122696)

[Table S1. Available syphilis self-test kits sold on e-commerce platforms in China^a^ 2](#_Toc101122697)

[Figure S1. Manufacturer-supplied step-by-step instructions 5](#_Toc101122698)

[Figure S2. Result report card 6](#_Toc101122699)

[Figure S3. Information Card 8](#_Toc101122700)

[Figure S4. Peer Notification Card 9](#_Toc101122701)

[Figure S5. Social network distribution 10](#_Toc101122702)

[Table S2. Baseline characteristics of study participants stratified by loss-to-follow-up in the Syphilis Self-Testing Randomized Controlled Trial in China. 11](#_Toc101122703)

[Table S3. Cost items included. 13](#_Toc101122704)

[Table S4. Sample size calculation 15](#_Toc101122705)

[Table S5 Break-down of adverse events among indexes at 3-month follow up survey 16](#_Toc101122706)

[Table S6 Break-down of adverse events among alters at alter survey 17](#_Toc101122707)

[Table S7. CONSORT 2010 checklist of information to include when reporting a randomised trial*. 18](#_Toc101122708)

[File S1 Study protocol 21](#_Toc101122709)

[Protocol Amendments 41](#_Toc101122710)

#

# Table S1. Available syphilis self-test kits sold on e-commerce platforms in China^a^

| **No.** | **Brand** | **Screenshot of the product** | **Approval** | **Quality assessment^b^** |
| --- | --- | --- | --- | --- |
| 1 | Wondfo (万孚) | 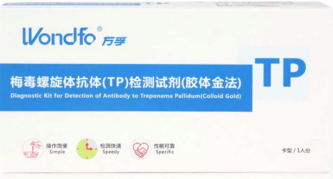 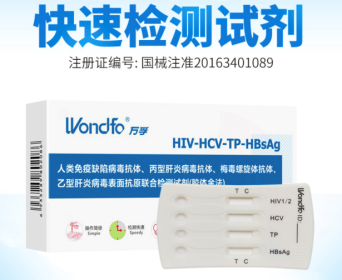 | CFDA | Sensitivity: 98.2%  Specificity: 98.9% |
| 2 | ALERE DETERMINE™ SYPHILIS TP | 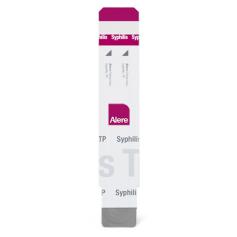 | CFDA, FDA (US) | Sensitivity: 96.5%  Specificity: 99.5% |
| 3 | Wan Tai (万泰) | 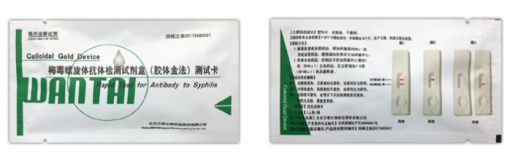 | CFDA | Sensitivity :98.2%  Specificity: 96.8% |
| 4 | ABON(艾博生物) | 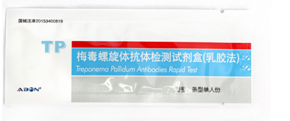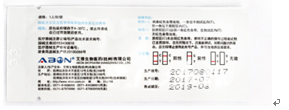 | CFDA | Sensitivity: 95.6%  Specificity: 93.1% |
| 5 | SD BIOLINE /Syphilis Duo HIV | 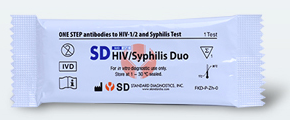 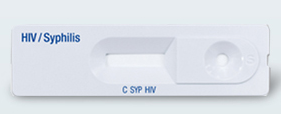 | CFDA  FDA | Sensitivity: 100%  Specificity: 99.1% |
|  | SD BIOLINE Syphilis 3.0 | 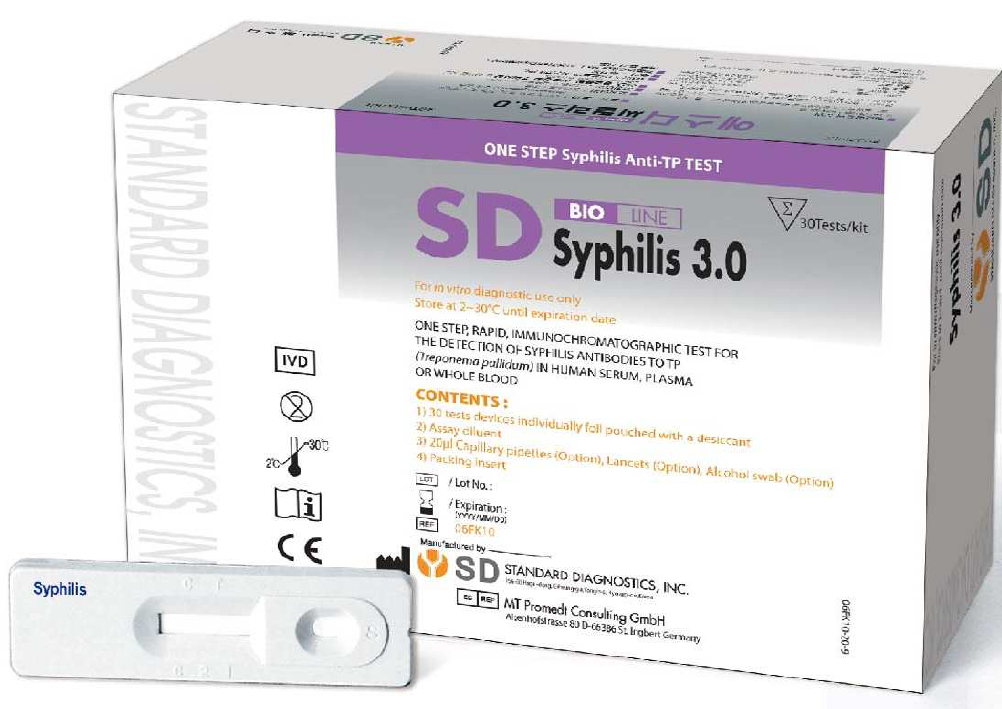 | FDA | Sensitivity: 99.3%  Specificity: 99.5% |
| 6 | David (大卫) | 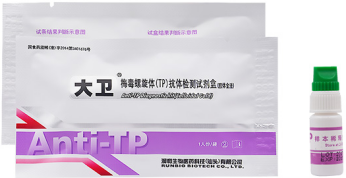 | CFDA | Sensitivity: 99.2%  Specificity: 99.9% |
| 7 | Accu News (准信) | 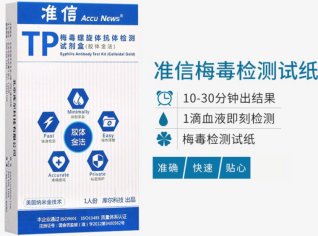 | CFDA | Sensitivity: 99.2%  Specificity: 100% |
| 8 | Nefertiti (奈費爾提蒂) | 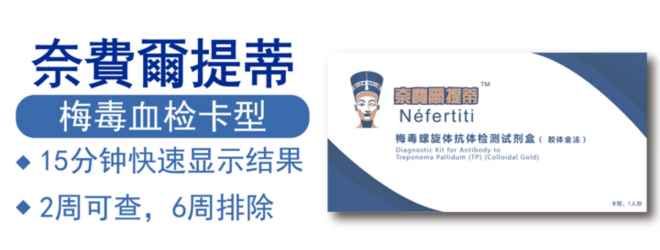 | CFDA | Sensitivity: 100%  Specificity:99.7% |
| 9 | Lavcae (爱威康) | 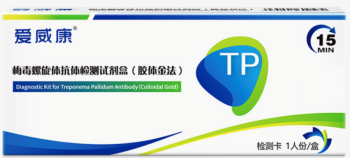 | CFDA | Sensitivity: 99.7%，  Specificity: 92.8% |
| 10 | Core tests | 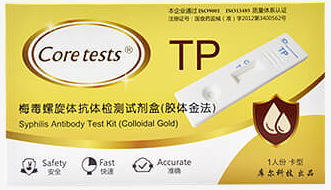 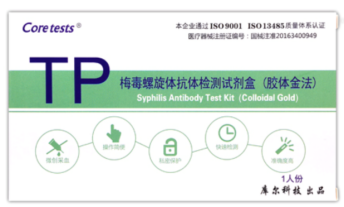 | CFDA | Sensitivity: 99.2%  Specificity: 100% |

^a^We searched on the first two largest e-commerce platforms in China: Taobao and Jingdong.

^b^The sensitivity and specificity results for the brand of number 1 to 5 are from national syphilis diagnostic reagent clinical evaluation report 2016. The results for the brand of number 6 to 10 are from each product’s official specification.

# Figure S1. Manufacturer-supplied step-by-step instructions


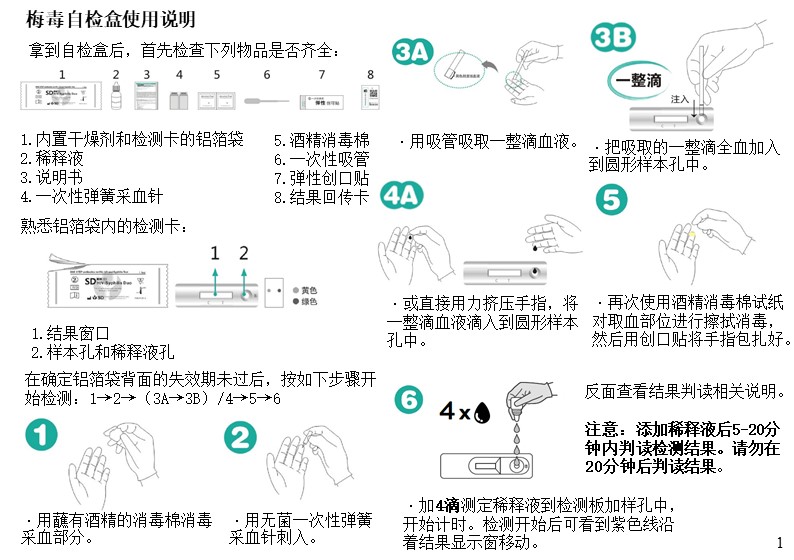


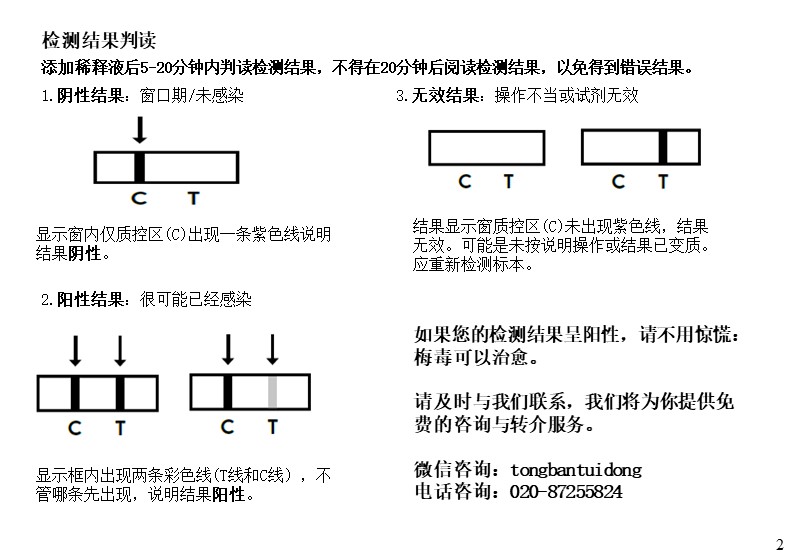


# Figure S2. Result report card


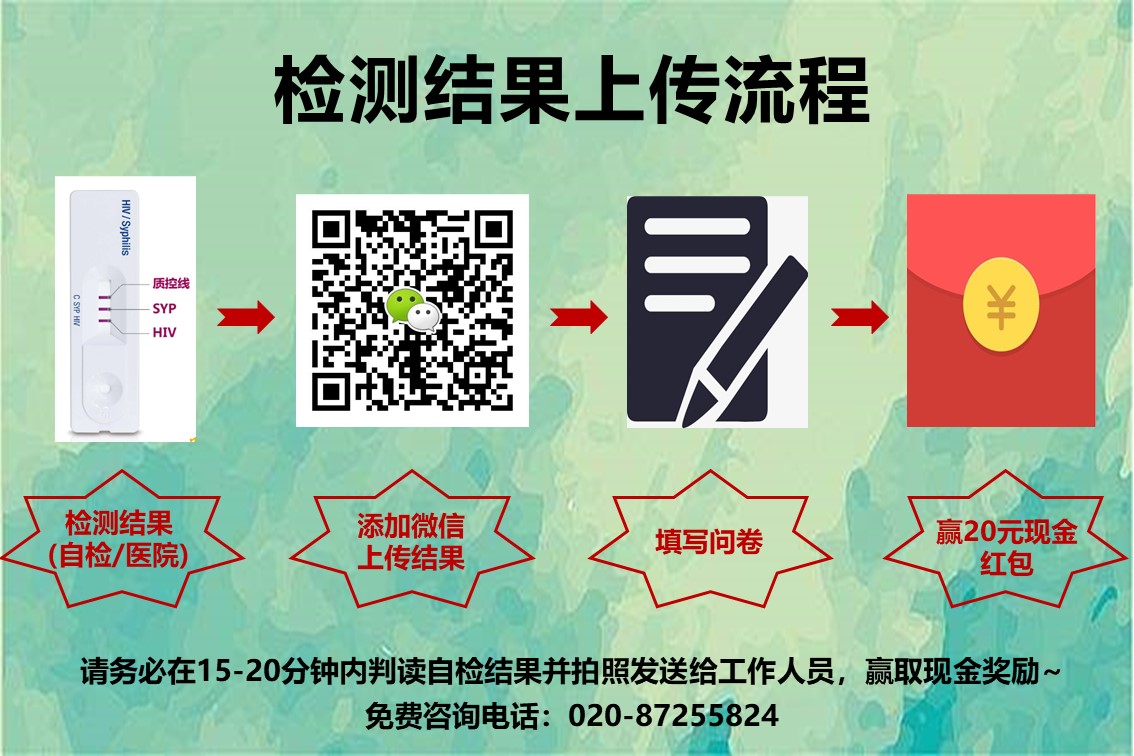


Procedure of verification of test results

Standard syphilis self-testing arm and Referral link syphilis self-testing arm

1. After performing syphilis self-testing, wait for the result.
2. Read the result within 20 minutes, take a picture of the plate.
3. Send a friend request by scanning the QR code, wait for research assistant’s (RA) approval.
4. Upload the picture of the test result along with the assigned number to RA’s Wechat account.
5. RA reads the result
   1. If it shows a negative result, research assistant confirms the result with the alter
   2. If it shows a positive result, alter would be referred to get confirmatory test in facility of their region.
6. RA sends the survey link to alter, then alter informed research assistant of the completion of the survey.
7. RA confirms the survey with alter, then send out the 20 RMB incentives.
8. RA renames the photo file according to the index number and returned order, then stored it in a encrypted folder.
9. The end of the photo verification for these two arms.

Standard of care arm

1. Get syphilis test at facility, wait for the result.
2. Take a picture of the test result sheet.
3. Send a friend request by scanning the QR code, wait for research assistant’s (RA) approval.
4. Upload the picture of the test result along with the assigned number to RA’s Wechat account.
5. RA reads the result
   1. If it shows a negative result, research assistant confirms the result with the alter
   2. If it shows a positive result, alter would be referred to get confirmatory test in institutes of their region if they haven’t done so.
6. RA sends the survey link to alter, then alter informed research assistant of the completion of the survey.
7. RA confirms the survey with alter, then send out the 20 RMB incentives.
8. RA renames the photo file according to the index number and returned order, then stored it in an encrypted folder.
9. The end of the photo verification for the arm.

# Figure S3. Information Card


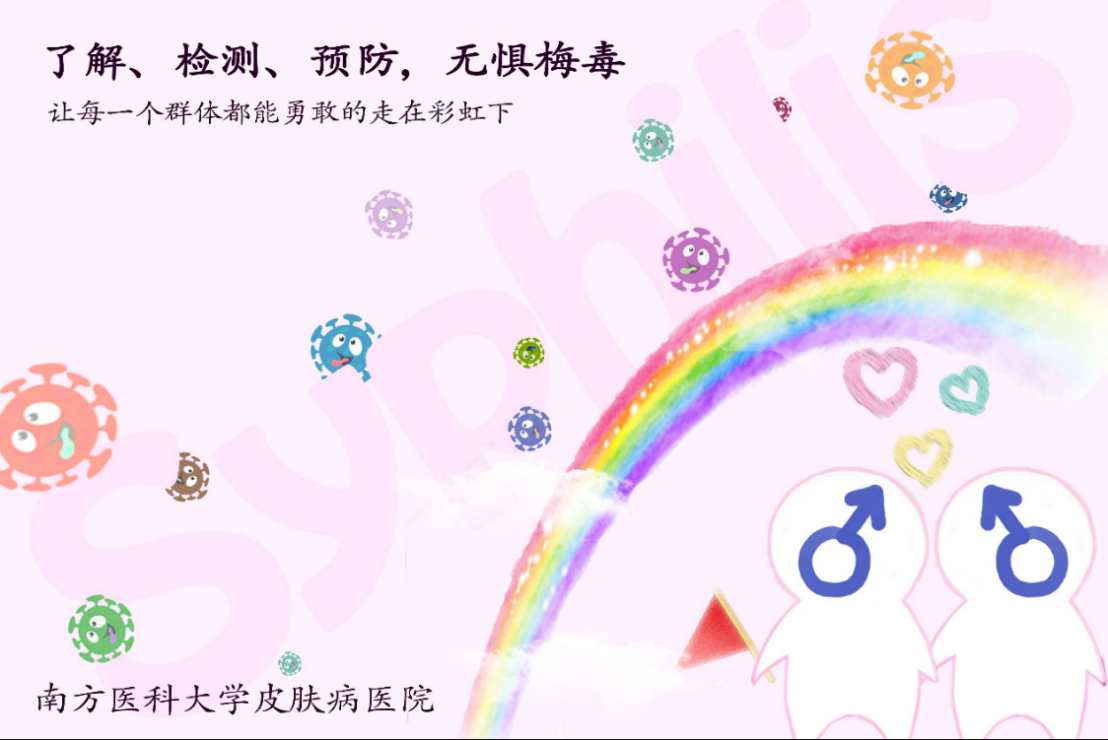


Front - The design of information card was crowdsourced. This is a runner-up design.


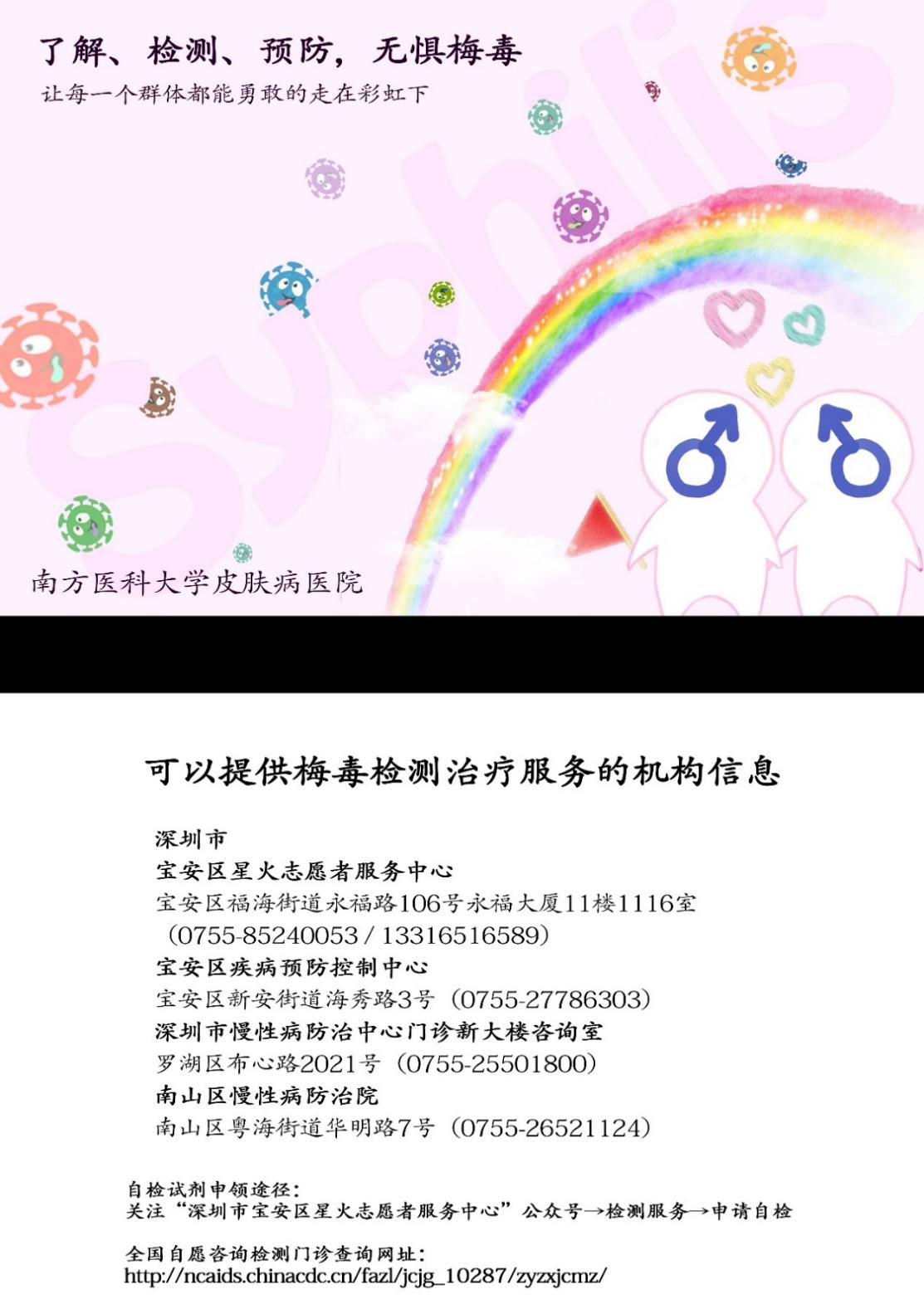


Back – This contains information about local syphilis testing services, which would be given at different study sites.

# Figure S4. Peer Notification Card


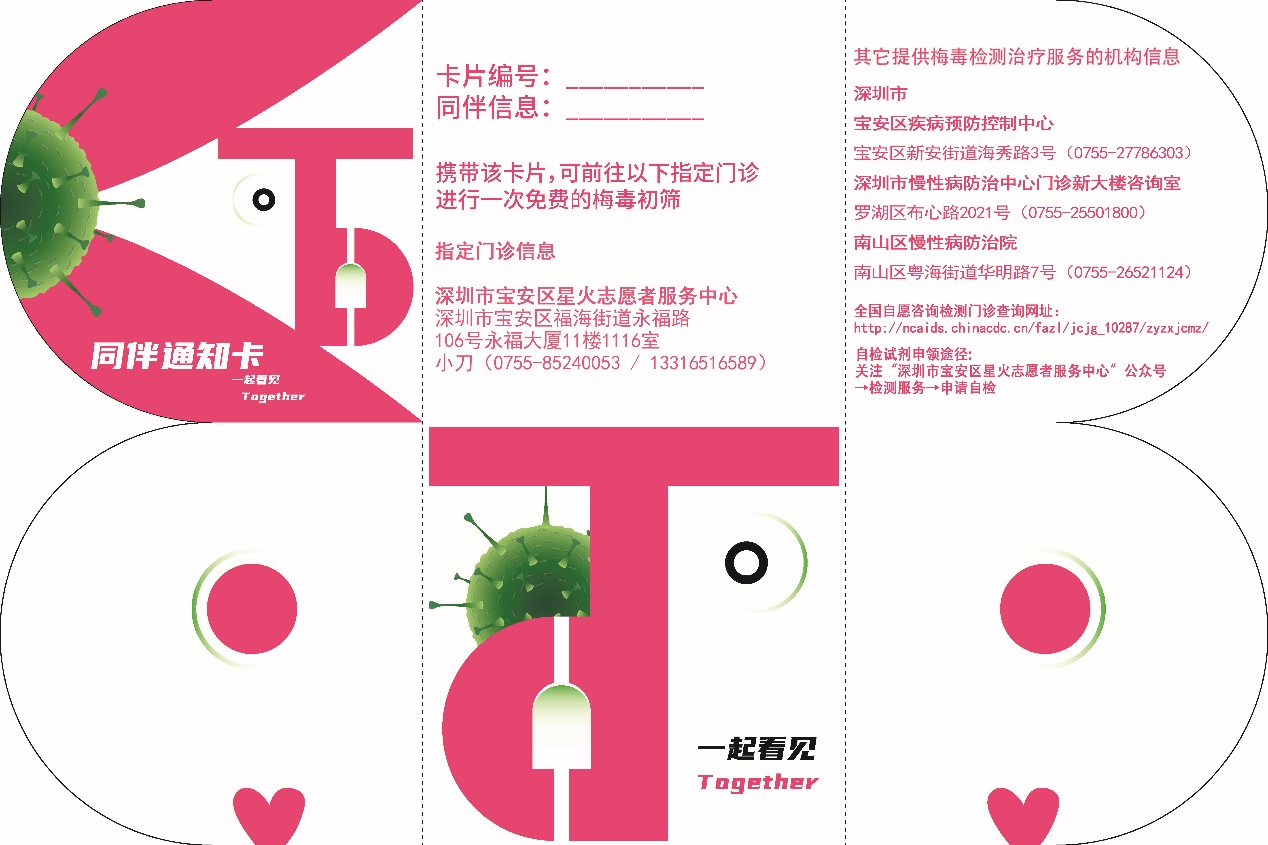


The design of the peer notification card was crowdsourced. This is the wining design.

# Figure S5. Demonstration on the social network of peer distribution


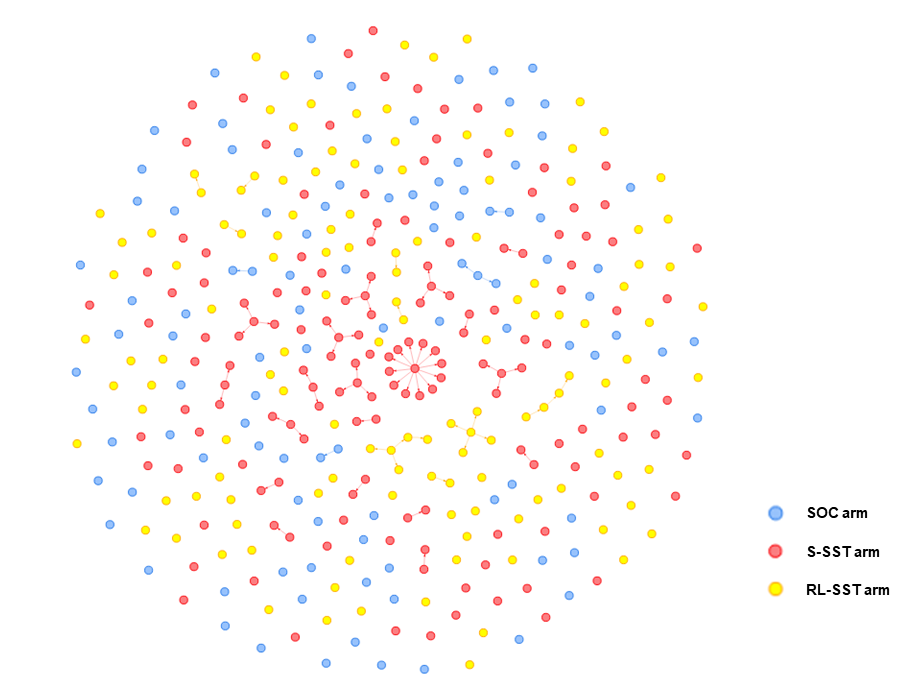


Please see following website for a high-definition version of the figure:

# Table S2. Baseline characteristics of study participants stratified by loss-to-follow-up in the Syphilis Self-Testing Randomized Controlled Trial in China.

|  | **Lost-to-follow-up (n=60)^a^** | **Completed follow-up survey (n=240)^a^** | ***p*-value^b^** |
| --- | --- | --- | --- |
| **Age (years)** | | | 0.603 |
| ≤30 | 27/60(45.0) | 117/240(37.8) |  |
| >30 | 33/60(55.0) | 123/240(51.2) |  |
| **Marital status** | | | 0.496 |
| Ever married | 17/60(28.3) | 79/240(32.9) |  |
| Never married | 43/60(71.7) | 161/240(67.1) |  |
| **Highest education** | | | 0.119 |
| High school or below | 37/60(61.7) | 121/240(50.4) |  |
| College or above | 23/60(38.3) | 119/240(49.6) |  |
| **Annual income (USD, $)** | | | 0.575 |
| ≤ 5500 | 10/60(16.7) | 37/240(15.4) |  |
| 5501-15000 | 42/60(70.0) | 157/240(65.4) |  |
| >15000 | 8/60(13.3) | 46/240(19.2) |  |
| **Sexual Orientations Disclosure** | | | 0.262 |
| Yes | 37/60(80.0) | 206/240(85.8) |  |
| No | 12/60(20.0) | 34/240(14.2) |  |
| **Number of male partners in the past 3 months** | | | 0.092 |
| ≤1 | 39/60(65.0) | 127/240(52.9) |  |
| >1 | 21/60(35.0) | 113/240(47.1) |  |
| **Social network size** | | | 0.419 |
| ≤2 | 34/60(56.7) | 122/240(50.8) |  |
| >2 | 26/60(43.3) | 118/240(49.2) |  |
| **Ever tested for HIV** | | | 0.051 |
| Yes | 51/60(85.0) | 223/240(92.9) |  |
| No | 9/60(15.0) | 17/240(7.1) |  |
| **Ever self-tested for HIV** | | | 0.10114 |
| Yes | 24/51(47.1) | 133/223(59.6) |  |
| No | 27/51(52.9) | 90/223(40.4) |  |
| **Ever infected with HIV** | | | **<0.001***** |
| Yes | 14/60(23.3) | 18/240(7.5) |  |
| No | 46/60(76.7) | 222/240(92.5) |  |
| **Ever tested for syphilis** | | | 0.185 |
| Yes | 38/60(63.3) | 173/240(72.1) |  |
| No | 22/60(36.7) | 67/240(27.9) |  |
| **Ever self-tested for syphilis** | | | 0.650 |
| Yes | 13/38(34.2) | 66/173(38.2) |  |
| No | 25/38(65.8) | 107/173(61.8) |  |

^a^Data are n/N (%) unless otherwise indicated. ^b^*p*-value computed using a two-sided Fisher’s exact test.

# Table S3. Cost items included

Table S3 summarizes the unit costs for the economic model using a healthcare provider perspective with a time horizon of 3 months (i.e. the study duration).

| **Variable costs** | **Unit cost (USD)** |
| --- | --- |
| Packaging | 0.43 |
| Instruction booklet | 0.12 |
| Syphilis test kit | 2.32 |
| Blood taking needle | 0.07 |
| Woundplast | 0.01 |
| Alcohol pad | 0.01 |
| Kraft bag | 0.08 |
| Activity flow chart | 0.77 |
| Peer notification card | 0.62 |
| Information card | 0.05 |
| Result upload card | 0.05 |
| Listing page | 0.02 |
| Baseline questionnaire subsidy | 3.00 |
| Alter questionnaire subsidy | 3.00 |
| Follow-up questionnaire subsidy | 4.00 |
| Successful distribution subsidy for indexes | 3.00 |
| Delivery cost of syphilis kits | 2 |
| Personnel recruitment | 8 |
| Travel for recruitment | 120 |
| Pen | 0.15 |
| A4 paper | 0.08 |
| **Fixed costs** | **Per month (USD)** |
| Internet | 30.96 |
| Power expense | 7.74 |
| Property management fee | 12.38 |
| Telephone bill | 15.48 |
| Wenjuanxing platform rent for data collection | 37.15 |
| Human cost | 300 per person |
| Working area rental | 309.6 |
| Computers | 619.2 |
| Cellphone | 154.80 |
| Desks | 77.71 |
| Desk chairs | 42.41 |
| Start-up Costs | **Total cost (USD)** |
| Sites coordination | 232.20 |
| Personnel training | 92.88 |
| Pilot study | 2321.98 |
| Crowdsourced activities | 247.68 |

# Table S4. Sample size calculation

**Sample size**

| **SST and standard of care** | µ1 | 0.05 |
| --- | --- | --- |
|  | µ2 | 0.45 |
|  | Standard deviation | 0.8 |
|  | Alpha | 0.05 |
|  | Power | 0.85 |
|  | Total sample size per arm | 39 |
| **LINK and standard of care** | µ_1_ | 0.05 |
|  | µ_2_ | 0.45 |
|  | Standard deviation | 0.8 |
|  | Alpha | 0.05 |
|  | Power | 0.85 |
|  | Total sample size per arm | 73 |

We used an inequality test to calculate the sample size (PASS 15.0, NCSS, LLC). The details of the sample size calculation are shown in Table S4. Preliminary study results from the pilot RCT showed that the number of alters by an index was 0.05 through standard of care delivery, 0.60 through standard SST delivery, and 0.45 through SST link delivery. Based on the above data, we assumed that the variances of the three groups were equal with the same standard deviation of 0.8. Assuming alpha of 0.05, power of 0.85, lost to follow-up rate of 0.20, we will need a minimum of 92 indexes per arm. To integrate the sample size calculations and to consider practicality, the final sample size would be 100 indexes in standard SST delivery arm, 100 indexes in SST referral link delivery arm, and 100 indexes in standard of care arm, with a total cluster size of 300 participants.

# Table S5 Break-down of adverse events among indexes at 3-month follow up survey

| Self-reported adverse events among indexes | RL-SST  (n=42) | SOC  (n=37) | S-SST  (n=48) |
| --- | --- | --- | --- |
| Alters didn’t know the reason why he was given the SST package, link or notification card | 47.6% (20/42) | 35.1% (13/37) | 29.2% (14/48) |
| Alter felt being humiliated for being given the SST package, online link or notification card | 4.8% (2/42) | 10.8% (4/37) | 4.2% (2/48) |
| Alter felt being mistrusted for giving the SST package, online link or notification card | 26.2% (11 /42) | 27.0% (10 /37) | 14.6% (7/48) |
| Index received verbal abuse, e.g argument, being shouted at when giving the SST package, online link or notification card | 4.8% (2/42) | 6.3% (3/37) | 0.0% (0/48) |
| Index received physical abuse, e.g being pushed or pulled or beaten up when giving the SST package, online link or notification card | 2.4% (1/42) | 2.7% (1/37) | 0.0% (0/48) |
| Index forced alter into testing using threatening, violence or negligence | 0.0% (0/42) | 2.7% (1/37) | 0.0% (0/48) |
| Index was being alienated for giving the SST package, online link or notification card | 1.4% (6/42) | 10.8% (4/37) | 10.4% (5/48) |
| None of these above | 45.2% (19/42) | 48.6% (18/37) | 60.4% (29/48) |
| Total adverse events | 54.8% (23/42) | 51.3% (19/37) | 39.6% (19/48) |

# Table S6 Break-down of adverse events among alters at alter survey

| Self-reported adverse events among alters | RL-SST  (n=31) | SOC  (n=5) | S-SST  (n=46) |
| --- | --- | --- | --- |
| Alter didn’t know the reason why he was given the SST | 29.0% (9/31) | 20.0% (1/5) | 21.7% (10/46) |
| Alter felt being humiliated when received the SST package, link or notification card | 6.5% (2/31) | 0.0% (0/5) | 2.2% (1/46) |
| Alter felt being mistrusted when received the SST package, link or notification card | 3.2% (1/31) | 0.0% (0/5) | 8.7% (4/46) |
| Alter had verbal conflict, e.g had an argument or shout at indexes when received the SST package, link or notification card | 3.2% (1/31) | 0.0% (0/5) | 2.2% (1/46) |
| Alter had physical conflict, e.g push or pull or beat up when received the SST package, link or notification card | 6.5% (2/31) | 0.0% (0/5) | 4.3% (2/46) |
| Alter being forced into testing using threatening, violence or negligence | 0.0% (0/31) | 0.0% (0/5) | 4.3% (2/46) |
| Index was being alienated for giving the SST package, online link or notification card | 0.0% (0/31) | 0.0% (0/5) | 2.2% (1/46) |
| None of these above | 71.0% (22/31) | 80.0% (4/5) | 73.9% (34/46) |
| Total adverse events | 29.0% (9/31) | 20.0% (1/5) | 26.1% (12/46) |

# Table S7. CONSORT 2010 checklist of information to include when reporting a randomised trial*.

| Section/Topic | Item No | Checklist item | Reported on page No |
| --- | --- | --- | --- |
| Title and abstract | | | |
|  | 1a | Identification as a randomised trial in the title | 1 |
|  | 1b | Structured summary of trial design, methods, results, and conclusions (for specific guidance see CONSORT for abstracts) | 3-4 |
| Introduction | | | |
| Background and objectives | 2a | Scientific background and explanation of rationale | 6-7 |
|  | 2b | Specific objectives or hypotheses | 7 |
| Methods | | | |
| Trial design | 3a | Description of trial design (such as parallel, factorial) including allocation ratio | 7 |
|  | 3b | Important changes to methods after trial commencement (such as eligibility criteria), with reasons | NA |
| Participants | 4a | Eligibility criteria for participants | 8 |
|  | 4b | Settings and locations where the data were collected | 7 |
| Interventions | 5 | The interventions for each group with sufficient details to allow replication, including how and when they were actually administered | 9-10 |
| Outcomes | 6a | Completely defined pre-specified primary and secondary outcome measures, including how and when they were assessed | 12 |
|  | 6b | Any changes to trial outcomes after the trial commenced, with reasons | NA |
| Sample size | 7a | How sample size was determined | 12 |
|  | 7b | When applicable, explanation of any interim analyses and stopping guidelines | NA |
| Randomisation: |  |  |  |
| Sequence generation | 8a | Method used to generate the random allocation sequence | 8-9 |
|  | 8b | Type of randomisation; details of any restriction (such as blocking and block size) | 8-9 |
| Allocation concealment mechanism | 9 | Mechanism used to implement the random allocation sequence (such as sequentially numbered containers), describing any steps taken to conceal the sequence until interventions were assigned | 8-9 |
| Implementation | 10 | Who generated the random allocation sequence, who enrolled participants, and who assigned participants to interventions | 8-9 |
| Blinding | 11a | If done, who was blinded after assignment to interventions (for example, participants, care providers, those assessing outcomes) and how | 8-9 |
|  | 11b | If relevant, description of the similarity of interventions | NA |
| Statistical methods | 12a | Statistical methods used to compare groups for primary and secondary outcomes | 12-13 |
|  | 12b | Methods for additional analyses, such as subgroup analyses and adjusted analyses | 12 |
| Results | | | |
| Participant flow (a diagram is strongly recommended) | 13a | For each group, the numbers of participants who were randomly assigned, received intended treatment, and were analysed for the primary outcome | 14 |
|  | 13b | For each group, losses and exclusions after randomisation, together with reasons | 14 |
| Recruitment | 14a | Dates defining the periods of recruitment and follow-up | 14 |
|  | 14b | Why the trial ended or was stopped | NA |
| Baseline data | 15 | A table showing baseline demographic and clinical characteristics for each group | 28 |
| Numbers analysed | 16 | For each group, number of participants (denominator) included in each analysis and whether the analysis was by original assigned groups | 14 |
| Outcomes and estimation | 17a | For each primary and secondary outcome, results for each group, and the estimated effect size and its precision (such as 95% confidence interval) | 14-16 |
|  | 17b | For binary outcomes, presentation of both absolute and relative effect sizes is recommended | 14-16 |
| Ancillary analyses | 18 | Results of any other analyses performed, including subgroup analyses and adjusted analyses, distinguishing pre-specified from exploratory | 15 |
| Harms | 19 | All important harms or unintended effects in each group (for specific guidance see CONSORT for harms) | 16 |
| Discussion | | | |
| Limitations | 20 | Trial limitations, addressing sources of potential bias, imprecision, and, if relevant, multiplicity of analyses | 19 |
| Generalisability | 21 | Generalisability (external validity, applicability) of the trial findings | 18 |
| Interpretation | 22 | Interpretation consistent with results, balancing benefits and harms, and considering other relevant evidence | 16-19 |
| Other information | | |  |
| Registration | 23 | Registration number and name of trial registry | 13 |
| Protocol | 24 | Where the full trial protocol can be accessed, if available | Appendix p21 |
| Funding | 25 | Sources of funding and other support (such as supply of drugs), role of funders | 13 |

* This checklist was retrieved from www.consort-statement.org

# File S1 Study protocol

**Social network distribution of syphilis self-testing among men who have sex with men in China: study protocol for a cluster randomized control trial**

Yajie Wang^1,2,3^*, Wei Zhang^4^*, Dongping Bao^2^, Jason J. Ong^5,6^, Joseph D. Tucker^4,6,7^, Rouxuan Ye^8^, Cheng Wang^1,2,3^

1. Dermatology Hospital of Southern Medical University, Guangzhou, Guangdong, China

2. Southern Medical University Institute for Global Health and Sexually Transmitted Diseases, Guangzhou, Guangdong, China

3. Guangdong Provincial Center for Skin Disease and STI Control, Guangzhou, Guangdong, China

4. University of North Carolina at Chapel Hill, Project-China, Guangzhou, Guangdong, China

5. Faculty of Infectious and Tropical Diseases, London School of Hygiene and Tropical Medicine, London, UK

6. Central Clinical School, Monash University, Victoria, Melbourne, Australia

7. Institute for Global Health and Infectious Diseases, School of Medicine, University of North Carolina at Chapel Hill, Chapel Hill, USA

8. Department of Biostatistics, Southern Medical University, Guangzhou, Guangdong, China

*These authors contributed equally to this work.

Corresponding to: Cheng Wang, PhD. Email: wangcheng090705@gmail.com

**Abstract**

**Background:** Syphilis is a common sexually transmitted infection (STI) among men who have sex with men (MSM). Increasing syphilis testing is important to syphilis control. However, in low- and middle-income countries like China, syphilis testing rates remain low among MSM. We describe a randomized controlled trial protocol to examine the effectiveness of social network distribution approaches of syphilis self-testing among MSM in China.

**Methods:** We will recruit index and alter MSM. Indexes will be eligible if they: are born biologically male; aged 18 years or above; ever had sex with another man; are willing to distribute syphilis testing packages or referral links to their alters; and willing to provide personal contact information for future follow-up. Three hundred MSM will be recruited and randomly assigned in a 1:1:1 ratio into three arms: standard of care (control arm); standard syphilis self-testing (SST) delivery arm; and referral link SST delivery arm. Indexes will distribute SST packages or referral links to encourage alters to receive syphilis testing. All indexes will complete a baseline survey and a 3-month follow-up survey. Syphilis self-test results will be determined by photo verification via a digital platform. The primary outcome is the mean number of alters who returned verified syphilis testing results per index in each arm.

**Discussion:** The trial findings will provide practical implications in strengthening syphilis self-testing distribution and increasing syphilis testing uptake among MSM in China. This study also empowers MSM community in expanding syphilis testing by using their own social network.

**Keywords:** Syphilis, Self-test, MSM, social network distribution, cRCT

**Background**

Syphilis remains a global health priority. The WHO estimated that the prevalence of syphilis among men who have sex with men (MSM) is 5% or more in at least 42 countries in 2018 [1]. From 2006-2012, the incidence of syphilis was 9.6 per 100 person-years among MSM living in China [2]. However, in low- and middle-income countries (LMICs) like China, syphilis testing rate is low among MSM. Studies showed that less than 30% of Chinese MSM has ever received a syphilis test [3].

Facility-based testing for syphilis has potential limitations related to stigma from providers [4], infrastructure requirement, inconvenience [5], and lack of privacy [6]. Recent advances in diagnostics such as self-testing, have enabled decentralized testing strategies [7] which makes testing more accessible. Syphilis self-testing is a process whereby an individual collects their own specimen, performs the test and interprets the result by themselves [3]. Several point-of-care syphilis tests have been approved and used for syphilis screening in China [8,9]. A cross-sectional study showed that syphilis self-testing among MSM could complement facility-based testing in China [3]. Approximately half of the MSM reported that syphilis self-testing was their first syphilis test, suggesting that this could expand test uptake among groups without a history of testing [3]. A recent randomized control trial reported that syphilis self-testing significantly increased syphilis testing among Chinese MSM with less cost per person tested compared to facility-based testing[10]. With the potential benefits of syphilis self-testing, further studies are needed to explore how this could be scaled up.

Social network distribution can be an effective syphilis self-testing distribution strategy to expand syphilis testing and to reach high-risk populations with undiagnosed syphilis. This strategy allows participants (defined as indexes) to apply for multiple self-testing kits and distribute to their peers (sexual partners or non-sexual partners, defined as alters) within their social network [11,12]. Social network distribution has been widely used for sexual partner notification with notification cards, which helps contain the spread of sexually transmitted infections (STIs) [13,14]. However, this strategy is likely to reach only a proportion of contacts. For example, it is more successful in reaching partners in long-term relationships and less so in reaching short-term casual sexual contacts [14,15]. Social network distribution of self-testing may be an effective approach to overcome these barriers [16]. A study showed that social network testing enables people at high risk for HIV or people living with HIV to encourage people in their social network to test for HIV [17]. This strategy is highly acceptable among MSM because it is distributed by trusted community leaders [18]. Social network distribution may reduce health disparities by decreasing barriers such as stigma and discrimination in health facilities [19], concerns for lack of confidentiality [20], and fear of sexuality disclosure [21] among hard-to-reach populations [17]. Given the feasibility of this strategy in expanding HIV self-testing among MSM, there is value in exploring whether this distribution strategy could also expand syphilis self-testing.

This study aims to examine the effectiveness of social network distribution approaches of syphilis self-testing through a three-arm cluster randomized controlled trial (cRCT) in Guangdong, China and to explore which approach could increase syphilis testing among Chinese MSM.

**Methods**

***Study design***

This is a non-blinded and paralell three-arm cluster randomized controlled trial among MSM living in China. Enrolled indexes will be randomly assigned in a 1:1:1 ratio into three arms: standard of care arm (control arm); standard SST delivery arm; and referral link SST delivery arm (referral link is used to apply for free SST packages). Indexes in the control arm will receive information packages with peer notification cards to encourage their alters to take a free syphilis screening at a designated health facility. Indexes in the standard SST delivery arm will receive free packages containing a SST kit to distribute to their alters. Indexes in the referral link SST delivery arm will be provided with SST referral links to distribute to their alters to access free syphilis self-testing packages online. Indexes in each arm will be followed up for three months (Figure 1). The hypothesis in this study is that standard and referral link syphilis self-testing social network delivery model are more effective than standard of care among Chinese MSM. Preliminary data from pilot study will be used to inform the final trial design. Preliminary pilot results are available on the Supplement materials.

***Study setting and population***

All indexes will be recruited by health workers at local MSM voluntary counselling and testing (VCT) clinics (in Foshan and Dongguan) or community-based organizations (CBOs) (in Shenzhen) from three cities in Guangdong Province, China. All sites are run by MSM community-based organizations (Xinghuo LGBT center, Shenzhen; Friends Care Center, Foshan; and Rainbow Center, Dongguan) and provide free HIV/STI screening and consultations for MSM. The staff at each site has rich experience with counselling, blood collection, rapid testing for syphilis, results reporting, and follow up of syphilis tests. All sites will follow the same study procedures.

Indexes will be eligible if they are: born biologically male; aged 18 years or above; ever had sex with another man; are willing to distribute syphilis testing packages or referral links to their alters; and willing to provide personal contact information for future follow-up. All participants need to sign an electronic informed consent before they fill out the baseline survey. Considering MSM living with HIV might be at higher risk of syphilis, we increase awareness of syphilis for MSM with HIV and mobilize health workers to include as many indexes with HIV as possible in the recruitment stage.

***Arms and interventions***

Arms and interventions are summarized in Table 1.

**Standard of care arm (control arm)**: Each index MSM will initially receive three information packages to distribute to their alters after completing their baseline survey. Each information package contains: 1) a health promotion and linkage to care information card; 2) a crowdsourced peer notification card; and 3) a syphilis testing result report card. Crowdsourcing is a practice in which a group solves a problem and then shares the solutions with the community [22]. Peer notification contains healthcare provider location and index information, which each alter can use to receive a free syphilis screening in a list of nearby facilities for each study site. Each alter can use a maximum of one package and take one free facility-based syphilis testing service through peer notification card from each index.

**Standard SST delivery arm**: Index MSM in this arm will initially receive three SST packages to distribute to their alters after completing their baseline survey. Different from the information package for the control arm, we will give them SST kits instead of the peer notification card. Each SST kit contains equipment for blood sample collection, quick syphilis test and a step-by-step pictorial instruction for using the self-test kit (shown in Supplementary material). Each alter can use a maximum one SST package and take syphilis self-testing service from each index. In this trial, we will use the syphilis SD Bioline Syphilis 3.0 rapid test kit). The sensitivity and specificity for syphilis range 85.7-100% and 95.5-99.4% respectively[16,23].

**Referral link SST delivery arm**: Index MSM in this arm will initially receive a unique SST referral link, which can be shared with up to three alters after baseline survey. Each link will expire after three times of usage. Each alter can only apply for one SST package with the link, which can be accessed by only one device (either a Wechat account or a phone number). Further, the SST package will be posted to the alter free of charge (free express delivery).

In all three study arms, each package or link will be assigned with a unique number for future returned results tracking and index matching. Alters will receive 3 USD when they send a photo verification of their self-testing or facility-testing results and complete the alter survey. Their matching index will also receive an extra 3 USD as incentive of successful distribution. In addition, each index can apply for additional three packages (refundable 4 USD deposit for each SST package; free of charge for notification card package) or for additional referral links when their initial link has been used by three alters returning their test results. Further, each alter in all three arms who returned his result can become a "secondary index" if he was born biologically male, aged 18 years or above, and ever had sex with another man. He can also apply for three packages (refundable 4 USD deposit for each SST package; free of charge for information package) or referral link to distribute to his alters. For all indexes and alters in the study, researchers will provide a 24/7 hotline and online counselling.

***Randomization and allocation***

At each site, eligible indexes will be assigned to one of the three arms through a cluster randomization procedure with ten participants per cluster. To ensure equal number of clusters in each of the three arms, the clusters will be randomized in a block of three at each study site. Computer-generated randomization codes will be produced and kept by a biostatistician who is not involved in participant enrollment. Ten blocks will be generated in total. Each block has three clusters consisting of standard of care arm, standard SST delivery arm, and referral link SST delivery arm, randomly created from all possible permutations. The schedule will be determined before the start of the study and then provided to each site. Each eligible index who presents to the site during a given 10-person cluster will be provided the allocated program.

***Blinding***

As it would not be possible to blind participants to their study arms, this is a non-blinded study. Investigators and staff assessing the outcomes will not be blinded to participants’ group assignments.

***Follow up***

The follow-up survey will be administered three months after recruitment for indexes. Each index will receive 4 USD after completing the follow-up survey. Any alters with a reactive self-testing results will be referred to undergo free confirmatory laboratory testing and clinical examination at study clinics or other local designated clinics/hospitals. If the participants are diagnosed with syphilis, we will undertake further follow-up to obtain treatment information. Alters with a reactive result who also return photo verification of confirmatory testing results or treatment proof will receive a further 3 USD. Alters with reactive results who do not return any proof for confirmation testing or treatment will be asked about further linkage to care information at the end of study.

***Outcome measures***

**Primary outcome**: Mean number of alters who returned photo verified syphilis testing results per index in each arm, including facility-based test and self-testing in 3 months.

**Secondary outcomes**: 1) Proportion of first-time syphilis testing among participants; 2) Proportion of testers with a positive syphilis testing result; 3) Economic evaluation in terms of total cost of implementing each of the three arms, the average cost per tester, the average cost per syphilis diagnosis, and the incremental cost-effectiveness ratio for the three arms; 4) Adverse events during the delivery procedures in each arm; 5) Mean number of alters who received the test kit; 6) Proportion of repeat testing among indexes and alters in each arm.

***Data collection***

**Syphilis testing record and results**

All indexes’ information will be recorded using online surveys (Wenjuanxing online survey platform). Alters can photograph and upload their testing results to an online social media platform “WeChat” (instant messaging service/application) by scanning the QR code in the result report card. Syphilis testing results can be either from facility-based testing or self-testing.

***Surveys***

**Baseline survey for index**

All eligible indexes will complete a baseline survey online on the web-based survey platform Wenjuanxing (Changsha Haoxing Information Technology Co., Ltd., China) at enrollment. The baseline survey collects information on socio-demographic information, history of sexual behaviors, syphilis testing, HIV testing and other STIs testing, and their social network. Sociodemographic information includes age, biological sex, residence status, marital status, highest education, monthly income, sexual orientation, and whether they disclosed their sexual orientation with others. Sexual behaviors include sexual history with men and/or women, role during sex with men, condom use, type of sex partners, group sex, and drug use. Facility-based testing or self-testing history of syphilis, HIV and other STIs, and treatment experience will also be collected. A set of questions including a scale to measure social support, community leadership, and the size of their social network will be asked[24,25].

**Alter survey**

Each alter in the three arms will receive an online survey link after uploading their syphilis test results. The survey will be created on the Wenjuanxing platform and includes questions on the relationship between the indexes and the alters, sociodemographic information, sexual behavior, the experience of receiving syphilis testing packages or links, testing history of syphilis, HIV and other STIs, and social network. The experience of accepting syphilis testing packages or links includes the behavior of the indexes when delivering syphilis testing packages or links, and the satisfaction to the delivery process of syphilis testing packages or links. Adverse events will be asked to identify whether the alters were being forced to test, have received physical and/or verbal abuse, or being misunderstood from indexes etc during the delivery procedures.

**Follow-up survey**

All indexes will complete a three-month follow-up survey online. The follow-up surveys will collect information of their experience of using the information packages, SST packages or referral link delivery; information on the relationship between the indexes and the alters; reasons of unwillingness to distribute; history of syphilis, HIV and other STIs testing in the past three months; and sexual behaviors in the past three months. Potential adverse events such as forced alters to test, received physical and/or verbal abuse, or being misunderstood from alters etc during the delivery procures are asked.

***Cost data***

A cost collection sheet will be used to identify, value and measure costs from the perspective of the program provider. This will include the start-up costs (e.g. packaging, peer notification card and result report card design, sites coordination, personnel training, pilot study), consumables (SST kits, standard of care testing supplies), capital costs (office equipment), and personnel costs. The time horizon will be the duration of the trial.

***Statistical methods***

**Sample size**

We used an inequality test to calculate the sample size (PASS 15.0, NCSS, LLC). The details of the sample size calculation are shown in Table 2. Preliminary study results from the pilot RCT showed that the number of alters by an index was 0.05 through standard of care delivery, 1.05 through standard SST delivery, and 0.60 through SST link delivery. Based on the above data, we assumed that the variances of the three groups were equal with the same standard deviation of 1. Assuming the number of clusters will be allocated 1:1:1 to each arm, with cluster size of 10 per cluster, alpha of 0.05, power of 0.9, inter class correlation of 0.01, lost to follow-up rate of 0.20, we will need a minimum of 10 clusters per arm. To integrate the sample size calculations and to consider practicality, the final sample size would be ten clusters in standard SST delivery arm, ten clusters in SST referral link delivery arm, and ten clusters in standard of care arm, with a total cluster size of 30 (300 participants).

**Table 2 Sample size calculations**

| **SST and standard of care** | ICC | 0.01 | 0.01 |
| --- | --- | --- | --- |
|  | Mean difference | 1 | 1 |
|  | Standard deviation | 1 | 1 |
|  | COV of cluster sizes | 0.65 | 0.65 |
|  | Number of people per cluster | 10 | 10 |
|  | Alpha | 0.05 | 0.05 |
|  | Power | 0.8 | 0.9 |
|  | Total sample size per arm | 20 | 30 |
|  | Number of clusters per arm | 2 | 3 |
| **LINK and standard of care** | ICC | 0.01 | 0.01 |
|  | Mean difference | 0.55 | 0.55 |
|  | Standard deviation | 1 | 1 |
|  | COV of cluster sizes | 0.65 | 0.65 |
|  | Number of people per cluster | 10 | 10 |
|  | Alpha | 0.05 | 0.05 |
|  | Power | 0.8 | 0.9 |
|  | Total sample size per arm | 60 | 80 |
|  | Number of clusters per arm | 6 | 8 |

ICC: Intra-class correlation; COV: Coefficient of variation.

***Data analysis***

**Primary analysis**

Sociodemographic characteristics will be summarized using descriptive statistics. The mean number of alters who returned photo verified syphilis testing result in each arm will be assessed using intention-to-treat analysis during the distribution period (three months). The effects of the intervention will be measured by comparing the mean number of alters who returned photo-verified syphilis testing results in the three arms by using generalized estimated equations modelling to account for potential correlations in outcomes within groups. Egocentric network analysis will be used to describe the network characteristics, such as network size (i.e., the total number of alters in index’s disclosure network)[24].

**Secondary analysis**

Within each arm of the study, we will calculate the proportion of first-time testers and testers with a positive syphilis testing result. Differences in proportions of first-time testers and testers with a positive syphilis testing result among alters during distribution period will be explored using Chi-square test and logistic regression in a multivariable model. We will also use descriptive analysis and chi-square test to compare the incidence of adverse events in three arms.

**Economic evaluation**

We will estimate the cost of syphilis testing in all three study arms. Costs will be categorized as fixed or variable costs. Fixed cost refers to cost that is independent of the number of tests conducted, including cost of start-up (see above), building rent and office equipment. Variable cost refers to cost that is dependent on the number of tests conducted, including SST kits, standard of care testing supplies, and personnel cost. Personnel costs will be calculated by multiplying the staff time associated with each program activity by the compensation received by the staff who perform these activities. We will first calculate the total cost for each group, then divide these costs by the number of participants tested and by the number of cases diagnosed with syphilis. For each arm of the study, we will calculate the incremental cost per person tested and the incremental cost per person diagnosed. To identify the optimal strategy, we will rank the incremental cost-effectiveness ratios of the three arms.

***Missing data plan***

We anticipate the loss to follow-up will be less than 20% in the 3-month follow up. If the primary outcome is missing for < 15% of participants, analyses will use a complete-case approach. If an outcome is missing for ≥15% of participants, missingness mechanism will be investigated and multiple imputation will be used if suitable.

**Discussion**

The prevalence of syphilis has remained high among MSM living in China while testing uptake is relatively low. A recent study demonstrated that using syphilis self-testing could increase the testing among this population[10]. However, there is limited evidence for the implementation of syphilis self-testing distribution models. In this study, we will test three syphilis testing models through social network distribution in a cluster randomized controlled trial. With the successful experience of HIVST secondary distribution and effectiveness of SST, we hypothesize that social network distribution of syphilis self-testing could increase the testing uptake among MSM in China.

This study examines the effectiveness of social network distribution on syphilis self-testing among MSM in China. Different from past studies on social network distribution, alters in our study could become secondary indexes. Alters of secondary indexes can become the next indexes as they wish. Our strategy may also empower MSM communities in expanding syphilis testing by using their own social networks. Participant recruitment will take place in facilities (community-based organization and specialist clinics), which offers better linkage to care for participants.

Several challenges might emerge in the implementation of this study. First, adverse events such as forced testing, physical and verbal abuse among participants, being misunderstood during the delivery procedures may occur. The findings of these questions will provide insights to design better syphilis self-testing support. Second, existing intervention delivery cannot guarantee all three packages or links are distributed. To encourage distribution, indexes will receive 3 USD for each successful distribution. In addition, we will try to understand why indexes fail to distribute any or partial packages via our survey. This information will help to improve current distribution models. Third, we ask for a refundable deposit in the SST arm if indexes wish to apply for SST packages again or secondary indexes wish to apply for SST packages. This might greatly discourage participation. For future studies, we need to discuss whether or not we should keep using this refundable deposit or adjust the amount if recruitment needs to be expanded. Lastly, though we used an objective measure for our primary outcome (photo verified syphilis testing results), there is a possibility that we underestimate the number of alters testing for syphilis if they don’t upload their testing results. We try to mitigate this by offering alters monetary incentives after their testing result is verified by a research assistant.

There are some limitations in this study. First, recruitment takes places in specialist hospitals and local CBOs. This will exclude individuals who are less likely to attend these recruitment sites, leading to sample bias. Second, we use data from treponemal test results, which might overlook testers’ treatment history and clinical data that help differentiate new and old cases.

In conclusion, this study will provide data on optimizing syphilis self-testing programs to increase syphilis testing uptake and earlier syphilis diagnosis among MSM in China.

**List of** **abbreviations**

CBO: Community-based organization; CEA: Cost-effectiveness analysis; cRCT: cluster randomized controlled trial; HIV: Human immunodeficiency viruses; HIVST: HIV self-testing; LGBT: Lesbian, gay, bisexual, and transgender; LMICs: Low- and middle-income countries; MSM: Men who have sex with men; POC: Point-of-Care; SST: Syphilis self-testing; STI: Sexually transmitted infection; VCT: Voluntary counselling and testing; WHO: World Health Organization.

**Ethical statement**

Institutional Review Board (IRB) approval has been obtained from Southern Medical University Guangdong Provincial Center for Skin Diseases and STI Control institutional ethical review board prior to study enrollment. All participants will provide consent prior to taking part in the study.

**Consent for publication**

Not applicable

**Availability of data and materials**

Not applicable.

**Competing interests**

The authors declare that they have no competing interests.

**Funding**

This study received support from the National Natural Science Foundation of China (81772240), Guangdong Medical Research Foundation (A2019402, A2019524). The funders had no role in study design, data collection, and analysis, decision to publish, or preparation of the manuscript. The authors thank all people who contributed to this study

**Authors' contributions**

WC, WYJ, ZW conceived the study. WYJ, ZW, BDP led pilot and implementation optimization. WYJ and YRX provided statistical expertise. WYJ, ZW, BDP recruited participants. WC, JT, JO provided oversight. WYJ, ZW and BDP wrote initial draft of the paper. WYJ and ZW revised and finalized the paper with inputs from WC, JT and JO. All authors read and authorized the final version.

**Acknowledgements**

We thank all study participants, CBO volunteers, Xinghuo LGBT Center, Friends Care Center, Rainbow Center, and Southern Medical University Guangdong Provincial Center for Skin Diseases and STI Control who contributed.

**Trial status**

At the time of writing this protocol, RCT recruitment and data collection are ongoing. Statistical analysis has not begun. The recruitment aims to conclude by December 15, 2020 and the follow-up will be finished by February 2021. The study has been registered with the Chinese Clinical Trial Registry (trial ID ChiCTR2000036988). Ethic approval has been obtained from Southern Medical University Dermatology Hospital. The trial protocol conforms to the Standard Protocol Items: Recommendation for Interventional Trials (SPIRIT) 2013 statement.

**Table 1 Group information**

| **Arm** | **Intervention** | **Intervention delivery** |
| --- | --- | --- |
| **Standard of care arm** | 1. **Information card**: the risk of acquiring syphilis, the importance of screening for syphilis, resources of three local syphilis test and linkage to care service sites, and a link of national syphilis VCT sites.  2. **Peer notification card**: social network distribution of notification card and encouragement of syphilis testing.  3. **Result report card** | 1. Each index will receive 3 information packages at enrollment, and distribute packages to 3 alters. Maximum **ONE** package per alter.  2. Each alter who returns his testing result and completes a survey can receive 3 USD and heir matched index will also receive an extra 3 USD.  3. Each index can apply for additional 3 packages when all his 3 alters return testing results.  4. Each eligible “secondary index” can apply for 3 information packages to distribute to his alters. |
| **Standard SST delivery arm** | 1. **Information card**: the risk of acquiring syphilis, the importance of screening for syphilis, resources of three local syphilis test and linkage to care service sites, and a link of national syphilis VCT sites.  2. **SST kit package**: social network distribution of SST kit package and encouragement of SST.  3. **Result report card** | 1. Each index will receive 3 SST packages at enrollment, and distribute packages to 3 alters. Maximum **ONE** package per alter.  2. Each alter who returns his testing result and completes a survey can receive 3 USD and heir matched index will also receive an extra 3 USD.  3. Each index can apply for additional 3 packages when all his 3 alters return testing results.  4. Each eligible “secondary index” can apply for 3 SST packages to distribute to his alters. |
| **Referral link SST delivery arm** | 1. **Information card**: the risk of acquiring syphilis, the importance of screening for syphilis, resources of three local syphilis test and linkage to care service sites, and a link of national syphilis VCT sites.  2. **SST referral link**: social network distribution of SST referral links and encouragement of SST.  3. **Result report card** | 1. Each index will receive 3 SST referral links at enrollment, and distribute links to 3 alters. Maximum **ONE** link per alter.  2. Each alter can apply for **ONE** SST package through the referral link, and SST package will be posted to him free of charge.  3. Each alter who returns his testing result and completes a survey can receive 3 USD and heir matched index will also receive an extra 3 USD.  4. Each index can apply for additional 3 referral links when all his 3 alters return testing results.  5. Each eligible “secondary index” can apply for 3 SST referral links to distribute to his alters. |

**Figure 1 Trial flow chat**

**3 MSM clinics in Guangdong**

**Inform consent**

**Eligibility screening and enrollment**

**10 MSM indexes per cluster**

**Cluster randomization** (1:1:1)

**Standard of care arm**

**(100 indexes)**

1. Information card

2. Peer notification card.

3. Result report card.

Apply for additional 3 packages when all his 3 alters return their testing results.

**Referral link SST delivery arm (100 indexes)**

1. Information card.

2. SST referral link.

3. Result report card.

Apply for additional 3 referral links when all his 3 alters return their testing results.

**Standard SST delivery arm (100 indexes)**

1. Information card.

2. SST kit package.

3. Result report card.

Apply for additional 3 packages (refundable deposit 4 USD/package) when all his 3 alters return their testing results.

**Enrollment**

**Allocation**

**Intervention**

**Baseline survey**

1. Each alter returns testing results and completes a survey, then receives 3 USD.

2. Each matched index receives 3 USD.

3.Each eligible “secondary index” can also apply for 3 information packages to distribute to their alters.

1. Each alter returns testing results and completes a survey, then receives 3 USD.

2. Each matched index receives 3 USD.

3.Each eligible “secondary index” can also apply for 3 SST packages (refundable deposit 4 USD/package) to distribute to their alters.

1. Each alter returns testing results and completes a survey, then receives 3 USD.

2. Each matched index receives 3 USD.

3.Each eligible “secondary index” can also apply for 3 referral links to distribute to their alters.

**Linkage to care**

1. Alters with positive SST results will be referred to undergo free confirmatory laboratory testing at study clinics or other local designated clinics.

2. Alters who diagnosed with syphilis will undertake one-month follow-up to obtain treatment information.

3. Each alter with reactive SST result who returns confirmatory laboratory testing results or returns treatment proof will receive 3 USD.

**Follow up**

**End follow-up survey for indexes (3 months)**

**References:**

1. Cheng W, Wang C, Tang W, Ong JJ, Fu H, Marks M, et al. Promoting routine syphilis screening among men who have sex with men in China: study protocol for a randomised controlled trial of syphilis self-testing and lottery incentive. BMC Infect Dis. 2020;20:455.

2. Chen G, Cao Y, Yao Y, Li M, Tang W, Li J, et al. Syphilis incidence among men who have sex with men in China: results from a meta-analysis. Int J STD AIDS. 2017;28:170–8.

3. Wang C, Cheng W, Li C, Tang W, Ong JJ, Smith MK, et al. Syphilis self-testing: A nationwide pragmatic study among men who have sex with men in china. Clin Infect Dis. 2020;70:2178–86.

4. Myers JE, El-Sadr WM, Zerbe A, Branson BM. Rapid HIV self-testing: long in coming but opportunities beckon. AIDS. 2013;27:1687–95.

5. Song Y, Li X, Zhang L, Fang X, Lin X, Liu Y, et al. HIV-testing behavior among young migrant men who have sex with men (MSM) in Beijing, China. AIDS Care. 2011;23:179–86.

6. Krause J, Subklew-Sehume F, Kenyon C, Colebunders R. Acceptability of HIV self-testing: a systematic literature review. BMC Public Health. 2013;13:735.

7. Ong JJ, Fu H, Smith MK, Tucker JD. Expanding syphilis testing: a scoping review of syphilis testing interventions among key populations. Expert Rev Anti Infect Ther. 2018;16:423—432.

8. WHO | The use of rapid syphilis tests. WHO. World Health Organization; 2014; https://www.who.int/reproductivehealth/publications/rtis/TDR_SDI_06_1/en/. Accessed 1 December 2020.

9. Weibin Cheng, Cheng Wang, Weiming Tang, Jason J. Ong, Hongyun Fu, Michael Marks, M. Kumi Smith, Changchang Li, Juan, Nie Peizhen Zhao, Heping Zheng, Bin Yang JDT. Promoting routine syphilis screening among men who have sex with men in China: a randomized controlled trial of syphilis self-testing and crowdsourced lottery incentive. BMC Infect Dis. 2020;1–8.

10. Wang C, Ong J, Zhao P, Tang W, Smith KM, Marks M, et al. Expanding syphilis test uptake using self-testing among men who have sex with men: a randomized controlled trial. Available at: https://ssrn.com/abstract=3718241

11. Lu Y, Ni Y, Li X, He X, Huang S, Zhou Y, et al. Monetary incentives and peer referral in promoting digital network-based secondary distribution of HIV self-testing among men who have sex with men in China: study protocol for a three-arm randomized controlled trial. BMC Public Health. 2020;20:911.

12. Wesolowski L, Chavez P, Sullivan P, Freeman A, Sharma A, Mustanski B, et al. Distribution of HIV Self-tests by HIV-Positive Men Who Have Sex with Men to Social and Sexual Contacts. AIDS Behav. 2019;23:893–9.

13. Bell G, Ward H, Day S, Ghani AC, Goan U, Claydon E, et al. Partner notification for gonorrhoea: A comparative study with a provincial and a metropolitan UK clinic. Sex Transm Infect. 1998;74:409–14.

14. Ward H, Bell G. Partner notification. Medicine. United Kingdom: Elsevier Ltd; 2014. p. 314–317.

15. Masters SH, Agot K, Obonyo B, Napierala Mavedzenge S, Maman S, Thirumurthy H. Promoting Partner Testing and Couples Testing through Secondary Distribution of HIV Self-Tests: A Randomized Clinical Trial. PLoS Med. 2016;13:e1002166.

16. Unemo M, Bradshaw CS, Hocking JS, de Vries HJC, Francis SC, Mabey D, et al. Sexually transmitted infections: challenges ahead. Lancet Infect. Dis. 2017; e235–79.

17. den Daas C, Geerken MBR, Bal M, de Wit J, Spijker R, Op de Coul ELM. Reducing health disparities: key factors for successful implementation of social network testing with HIV self-tests among men who have sex with men with a non-western migration background in the Netherlands. AIDS Care - Psychol Socio-Medical Asp AIDS/HIV. 2020;32:50–6.

18. Okoboi S, Lazarus O, Castelnuovo B, Nanfuka M, Kambugu A, Mujugira A, et al. Peer distribution of HIV self-test kits to men who have sex with men to identify undiagnosed HIV infection in Uganda: A pilot study. PLoS One. 2020;15:22–8.

19. Okoboi S, Twimukye A, Lazarus O, Castelnuovo B, Agaba C, Immaculate M, et al. Acceptability, perceived reliability and challenges associated with distributing HIV self-test kits to young MSM in Uganda: a qualitative study. J Int AIDS Soc. 2019;22:e25269.

20. Blondell SJ, Kitter B, Griffin MP, Durham J. Barriers and Facilitators to HIV Testing in Migrants in High-Income Countries: A Systematic Review. AIDS Behav. 2015;19:2012–24.

21. Deblonde J, De Koker P, Hamers FF, Fontaine J, Luchters S, Temmerman M. Barriers to HIV testing in Europe: a systematic review. Eur J Public Health. 2010;20:422–32.

22. Wang C, Han L, Stein G, Day S, Bien-Gund C, Mathews A, et al. Crowdsourcing in health and medical research: a systematic review. Infect Dis poverty. 2020;9:8.

23. Mabey D, Peeling RW, Ballard R, Benzaken AS, Galbán E, Changalucha J, et al. Prospective, multi-centre clinic-based evaluation of four rapid diagnostic tests for syphilis. Sex Transm Infect. 2006;82 Suppl 5:v13–6.

24. Cao B, Saffer AJ, Yang C, Chen H, Peng K, Pan SW, et al. MSM Behavior Disclosure Networks and HIV Testing: An Egocentric Network Analysis Among MSM in China. AIDS Behav. 2019;23:1368–74.

25. Wu D, Tang W, Lu H, Zhang TP, Cao B, Ong JJ, et al. Leading by Example: Web-Based Sexual Health Influencers Among Men Who Have Sex With Men Have Higher HIV and Syphilis Testing Rates in China. J Med Internet Res. 2019;21:e10171.

# Protocol Amendments

Protocol title: Social network distribution of syphilis self-testing among men who have sex with men in China: study protocol for a cluster randomized control trial

Revisions to V1.0: (Original protocol)

Date: V2.0: (Amendment 01)

| Change | Rationale | Affected Protocol Sections |
| --- | --- | --- |
| 1.Randomization was changed to block randomization | 1. To make the randomization at study sites easier and clearer.   In our study, we replaced the randomization from “cluster randomization” in the protocol with “block randomization”. This change makes randomization at study sites clearer for recruitment staff. Although we have made this change, we assigned eligible indexes into one of three arms 1:1:1.     1. To make the analysis more logical | Randomization <page 30> |
| 2.Statistical analysis was changed to negative binomial model. | In the original protocol, we mentioned that “…using generalized estimated equations modelling to account for potential correlations in outcomes within groups” During the data analysis stage, we found that data distribution is abnormal and skewed. The negative binomial regression is more inclusive considering the data distribution and provides flexibility to model overdispersion. | Primary analysis, secondary analysis, subgroup analysis  <Page 35> |
| 3.Sample size | Sample size calculation was changed due to the change of randomization. For new sample size calculation, please refer to Appendix page 13. | Sample size calculation <Page 36> |
